# The Rho/MRTF pathway inhibitor CCG-222740 reduces stellate cell activation and modulates immune cell populations in Kras^G12D^; Pdx1-Cre (KC) mice

**DOI:** 10.1038/s41598-019-43430-0

**Published:** 2019-05-08

**Authors:** Ana S. Leal, Sean A. Misek, Erika M. Lisabeth, Richard R. Neubig, Karen T. Liby

**Affiliations:** 1 Michigan State University, Department of Pharmacology & Toxicology, East Lansing, MI USA; 2 Department of Physiology, East Lansing, MI USA

**Keywords:** Cancer microenvironment, Drug development

## Abstract

The stromal reaction in pancreatic cancer creates a physical barrier that blocks therapeutic intervention and creates an immunosuppressive tumor microenvironment. The Rho/myocardin-related transcription factor (MRTF) pathway is implicated in the hyper-activation of fibroblasts in fibrotic diseases and the activation of pancreatic stellate cells. In this study we use CCG-222740, a small molecule, designed as a Rho/MRTF pathway inhibitor. This compound decreases the activation of stellate cells *in vitro* and *in vivo*, by reducing the levels of alpha smooth muscle actin (α-SMA) expression. CCG-222740 also modulates inflammatory components of the pancreas in KC mice (LSL-Kras^G12D/+^; Pdx-1-Cre) stimulated with caerulein. It decreases the infiltration of macrophages and increases CD4 T cells and B cells. Analysis of the pancreatic adenocarcinoma (PDA) TCGA dataset revealed a correlation between elevated RhoA, RhoC and MRTF expression and decreased survival in PDA patients. Moreover, a MRTF signature is correlated with a Th2 cell signature in human PDA tumors.

## Introduction

Pancreatic cancer is one of the most lethal cancers, with a 5-year survival rate below 10%^1^. Despite recent therapeutic advances, current treatment modalities for pancreatic cancer lack therapeutic efficacy, mostly due to an intense stromal reaction^2,3^. The stroma can account for as much as 90% of the tumor volume in pancreatic tumors^4,5^. Cancer associated fibroblasts (CAFs) are responsible for the production of matrix proteins that lead to enhanced microenvironment stiffness, and consequently therapeutic resistance^6^. Pancreatic stellate cells (PSCs), a type of fibroblast, are also present in the pancreas tumor microenvironment. PSCs support cancer cells by providing essential nutrients^7^ and by maintaining an immunosuppressive environment^8,9^, through excretion of essential amino acids and cytokines, respectively.

PSCs store lipids in the pancreas and are responsible for the turnover of the extracellular matrix (ECM)^10,11^. In a healthy pancreas these cells, which are 4–7% of the total mass of the pancreas, exist in a quiescent state and are characterized by abundant cytoplasmic lipid droplets rich in vitamin A^7,8,12,13^. During pancreatic injury, these cells are activated by cytokines, growth factors, or oxidative and metabolic stress, leading to differentiation to a myofibroblast-like cell state. Activated PSCs lose their cytoplasmic lipid droplets, express alpha-smooth muscle actin (α-SMA), a marker of fibroblast activation, acquire proliferative capacity, and increase synthesis of ECM proteins^7,14,15^. PSCs act cooperatively with pancreatic cancer cells, as pancreatic cancer cells produce mitogenic and fibrogenic factors, such as platelet-derived growth factor (PDGF), transforming growth factor β (TGF-β), and sonic hedgehog (SHH)^3,12^, that promote the activation of PSCs. Reciprocally, activated PSCs produce PDGF, insulin-like growth factor 1 (IGF1), connective tissue growth factor (CTGF), and other factors that may promote cancer cell proliferation, migration, and survival^7,12,15,16^.

Mechanoregulatory circuits between signaling pathways allow physical cues from the stromal ECM to control cell growth and tissue transformation, creating a mutual dependency^17–19^. The RhoA-C subfamily of Rho guanosine triphosphatases (Rho GTPases) regulates multiple biological functions; most notably, they stimulate the polymerization of G-actin into F-actin stress fibers. Through modulation of the actin cytoskeleton, Rho GTPases regulate gene transcription through modulation of myocardin-related transcription factor (MRTF)/serum response factor (SRF) activity. These important signaling GTPases also influence cell polarity, microtubule dynamics, membrane transport pathways and transcription factor activity^20^. GTPases are commonly hijacked in tumor cells and drive tumor progression.

Altering cytoskeleton-based cell contractility affects cancer cell invasion and also modulates interactions between stromal and cancer cells. This results in tissue stiffening driving tumor survival, proliferation, and progression. In fibroblasts, the Rho/MRTF pathway has classically been associated with cell contractility, and CAFs rely on this pathway to generate tracks for cancer cell migration. Amoeboid movement acquired by cancer cells following remodeling of the extracellular matrix by CAFs is also dependent on the Rho/MRTF pathway^21,22^.

Targeting tissue architecture via Rho GTPase inhibition with small molecules is an emerging area for potential therapeutic intervention in cancer. It can modulate tissue stiffness, cellular rheology, vasodilation and mechanoplasticity^17,18,23^. In order to target transcriptional pathways downstream of Rho, CCG-1423 was identified in a cell-based high throughput screen. Further optimization of the chemical scaffold led to the synthesis of CCG-222740, with increased potency, decreased *in vivo* toxicity, and increased *in vivo* half-life. This potent and selective MRTF inhibitor effectively reduces fibrosis in skin and blocks melanoma metastasis^22,24^. Despite the established role for Rho/MRTF signaling in stellate cells, the effects of inhibition of this transcriptional mechanism have yet to be determined in pancreatic cancer stroma formation. In PSCs fasudil, a Rho kinase (ROCK) inhibitor, decreases activation of these cells by reducing the production of α-SMA^25^. ROCK inhibition with fasudil also potentiates gemcitabine response, possibly through modulation of the tumor microenvironment and extracellular matrix composition^21^. These findings led us to hypothesize that the Rho/MRTF inhibitor CCG-222740 may be an effective approach to reduce the activation of stellate cells in the pancreas and consequently reduce the formation of fibroinflammatory stroma in the context of pancreatitis in a relevant mouse model for pancreatic cancer.

The development of pancreatic cancer is dependent on several oncogenic modifications. *Kras* is the most frequently mutated gene (G12D allele) in pancreatic cancer and is found in 95% of pancreatic cancers^26^. Although genetically engineered mouse (GEM) models have convincingly demonstrated that constitutive activation of *Kras* alone is sufficient for the initiation and progression of this disease, progression is accelerated when an inflammatory stimulus is added^27^. Chronic or repeated acute pancreatitis (inflammation of the pancreas) is a risk factor for the development of pancreatic cancer^28,29^. In this study we used *in vitro* and *in vivo* tools to study the effects of CCG-222740. For the *in vitro* studies, primary stellate cells isolated from the pancreas of wild type mice and immortalized CAFs isolated from the tumor of a pancreatic cancer GEM model induced by an activating *Kras* mutation^6^ were used. The *in vivo* studies were done in LSL-Kras^G12D/+^; Pdx-1-Cre (KC) mice stimulated with caerulein to induce pancreatitis. With these tools, we tested the efficacy of CCG-222740 for inhibiting the formation of stroma and the pathogenesis of pancreatic cancer.

## Results

### Pharmacologic inhibition of the Rho/MRTF pathway in PSCs and CAFs

**Figure 1:**
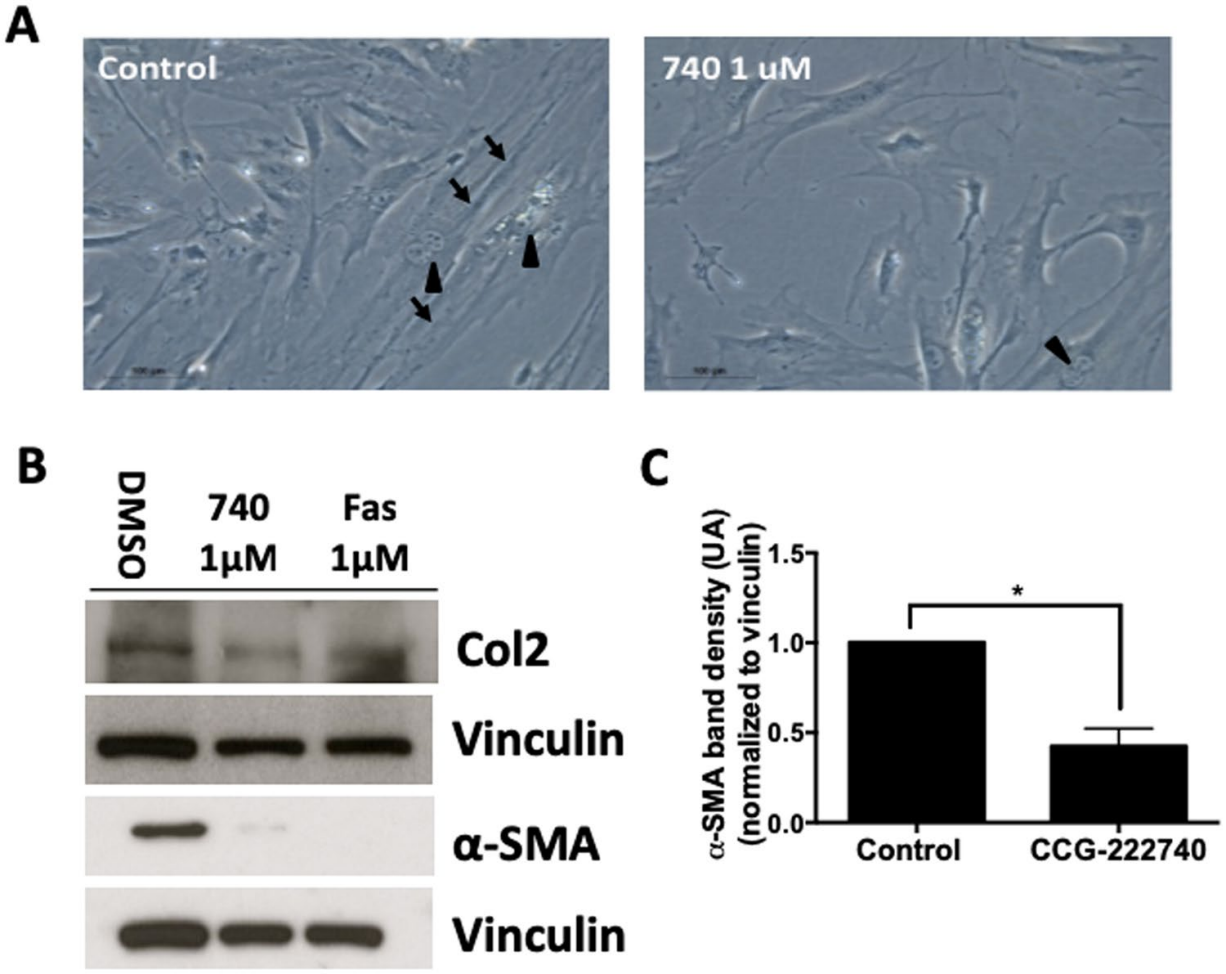
Treatment with the Rho/MRTF pathway inhibitor CCG-222740 prevents the activation of primary stellate cells. Stellate cells isolated from the pancreas of wildtype mice were treated with CCG-222740 at 1 μM for 6 days. (**A**) Bright field microscopy of the stellate cells; arrows point to fibers and arrowheads to duplicating cells (100x magnification). (**B**) Stellate cell activation was evaluated by measuring the levels of alpha smooth muscle actin (α-SMA) and collagen 2 A (COL2A) by Western blotting. (**C**) Levels of α-SMA were normalized to vinculin and quantified using ImageJ. Data are represented as mean ± SEM. Blots are representative of 3 independent experiments. Additional blots in Supplemental Fig. 8. *p < 0.05.

PSCs, isolated as previously described^10^ from the pancreas of wild type C57BL/6 mice, were cultured for 3 days to achive confluence and then treated with the Rho/MRTF pathway inhibitor CCG-222740 for 6 days. Once grown as a monolayer, the PSCs acquire a myofibroblast phenotype^14^. As shown in Fig. 1A, PSCs have an elongated shape and show multiple nuclei, consistent with cell duplication. The effects of the drug were further evaluated by western blot to determine the levels of α-SMA protein, a marker for stellate cell activation. Treatment with 1 μM of CCG-222740 significantly (p < 0.05) reduced the levels of α-SMA and collagen 2 A levels in the PSCs (Fig. 1B,C). Fasudil, a ROCK inhibitor previously reported to reduce the activation of stellate cells^25^, was also tested. Both CCG-222740 and fasudil reduced the levels of α-SMA and collagen II in PSCs (Fig. 1B).

**Figure 2:**
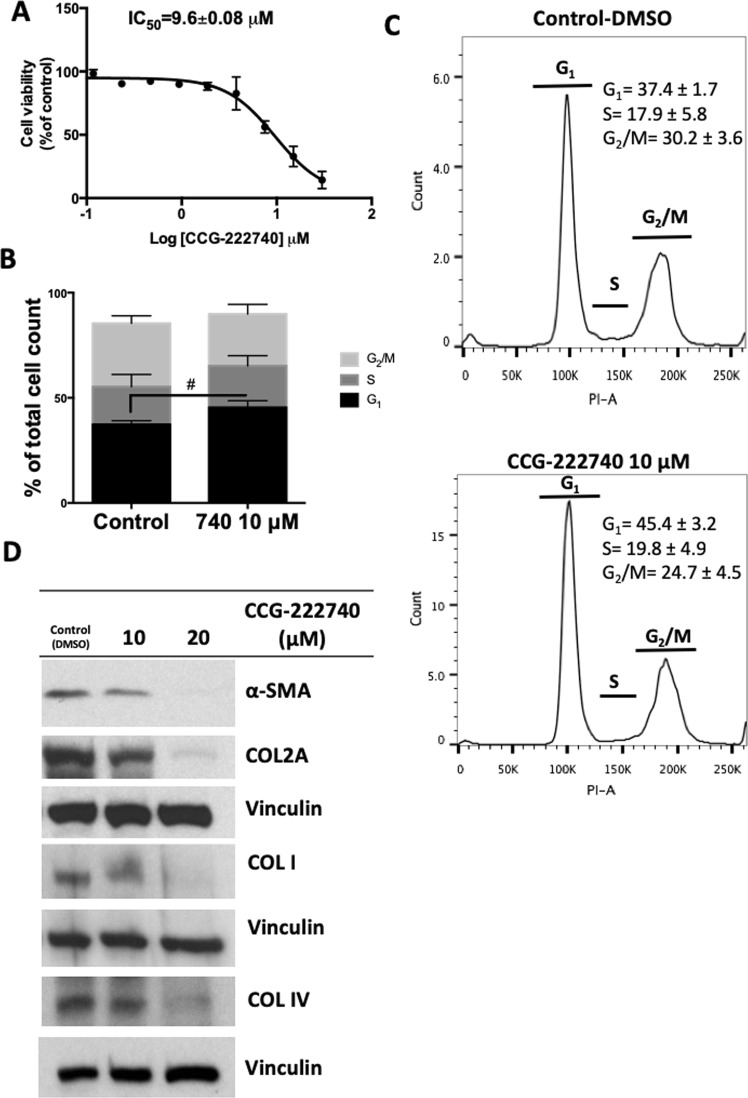
Treatment with the Rho/MRTF pathway inhibitor CCG-222740 reduces viability and collagen production in cancer associated fibroblasts (CAFs) from Kras LSL-G12D; Trp53 LSL-R172H; Pdx1-Cre (KPC) mice. (**A**) CAFs were treated with several concentrations of CCG-222740 for 72 hours, and cell viability was determined by an MTT assay. (**B**,**C**) Cell cycle analysis of CAFs treated with 10 µM CCG-222740 (740) for 72 hours; ^#^p = 0.09. (**D**) Protein levels of alpha smooth muscle actin (α-SMA), collagen 2 A (COL2A), collagen I (COL I) and collagen IV (COL IV) were determined in CAFs after treatment with CCG-222740 for 72 hours. Additional blots included in Supplemental Fig. 9.

CAFs isolated from a mouse pancreatic tumor induced by a *Kras* mutation^6^ were treated with the Rho/MRTF inhibitor CCG-222740 for 72 hours. CCG-222740 treatment decreased cell viability of CAFs, with an IC_50_ of ~10 μM, as measured by the MTT assay (Fig. 2A). CCG-222740 also had growth inhibitory effects on human and mouse pancreatic cancer cells, inducing growth arrest at similar concentrations as the CAFs (Sup Fig. 2). Although the induction of G1 cell cycle arrest in CAFs was not statistically significant (p = 0.09, Fig. 2B,C), CCG-222740 increased the protein levels of p27 and decreased cyclin D1 (Sup Fig. 1A)^22^. In pancreatic cancer, CAFs are one of the main producers of matrix proteins, including several collagen isoforms. The Rho/MRTF pathway inhibitor CCG-222740 decreased the levels of collagens I, 2a and IV, as well as α-SMA in the CAFs (Fig. 2D). Fasudil also reduced the levels of α-SMA in these cells, however, treatment at higher concentrations increased collagen I and 2a levels (Sup Fig. 1B).

In cancer cells, CCG-222740 treatment leads to the exclusion of MRTF from the nucleus^22^. CAFs treated with CCG-222740 showed a small accumulation of MRTF in the nucleus, and fasudil showed a slight decrease in the accumulation of this protein in the nucleus (Sup Fig. 1C). The difference between nuclear localization upon treatment with CCG-222740 in cancer cells versus CAFs might be because these cells have no genetic alterations that induce the pathway. Additionally, the targeting of MRTF in normal physiological contexts, such as in CAFs, might prompt the cells to actively translocate more protein to the nucleus to maintain physiological levels of activation.

### Rho/MRTF pathway inhibition in caerulein-stimulated KC mice suppressed PSC activation as determined by expression of α-SMA

**Figure 3:**
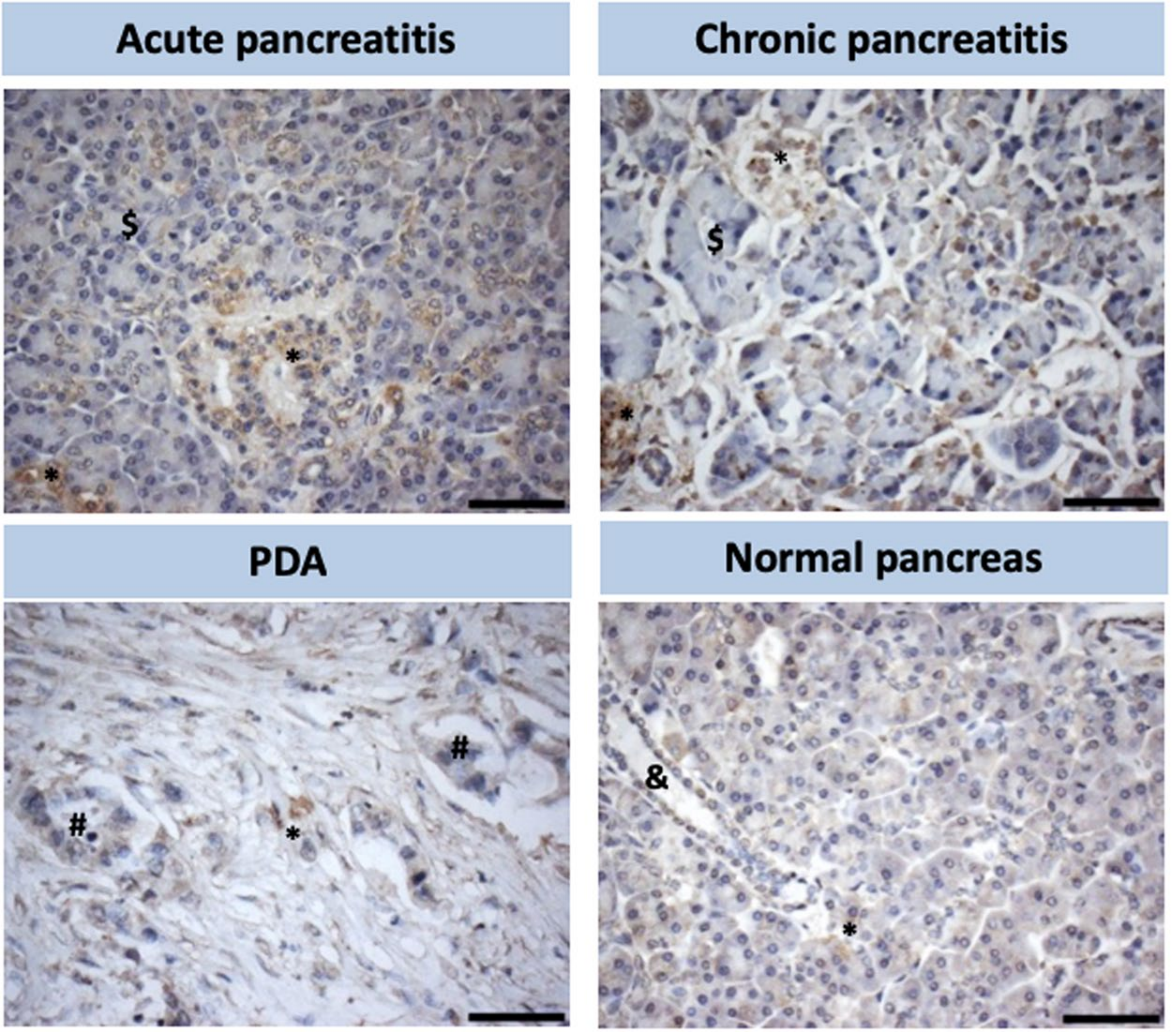
Stellate cell activation in various pathologies of the human pancreas. Immunohistochemical staining for α-SMA in representative tissue microarrays of acute and chronic pancreatitis, pancreatic ductal adenocarcinoma (PDA) and normal pancreas. *Activated stellate cells (α-SMA positive), ^$^reactive acinar cells, ^#^pancreatic adenocarcinoma cells, ^&^ normal pancreatic duct. Scale Bar: 60 μm.

Activated pancreatic stellate cells, which are positive for α-SMA, are enriched in patients with acute or chronic pancreatitis (Fig. 3). In human pancreatic cancer, α-SMA-positive cells are localized predominantly in the stroma, highlighting the presence of CAFs and stellate cells (Fig. 3). In healthy pancreas tissue, α-SMA-positive cells are rare, and α-SMA is typically only expressed in activated stellate cells, which maintain a homeostatic extracellular matrix (Fig. 3). Since pancreatitis is a risk factor for pancreatic cancer, the presence of high numbers of activated PSCs suggests these cells play a role in promoting pancreatic cancer progression by secreting cytokines and chemokines^7,15^.

**Figure 4:**
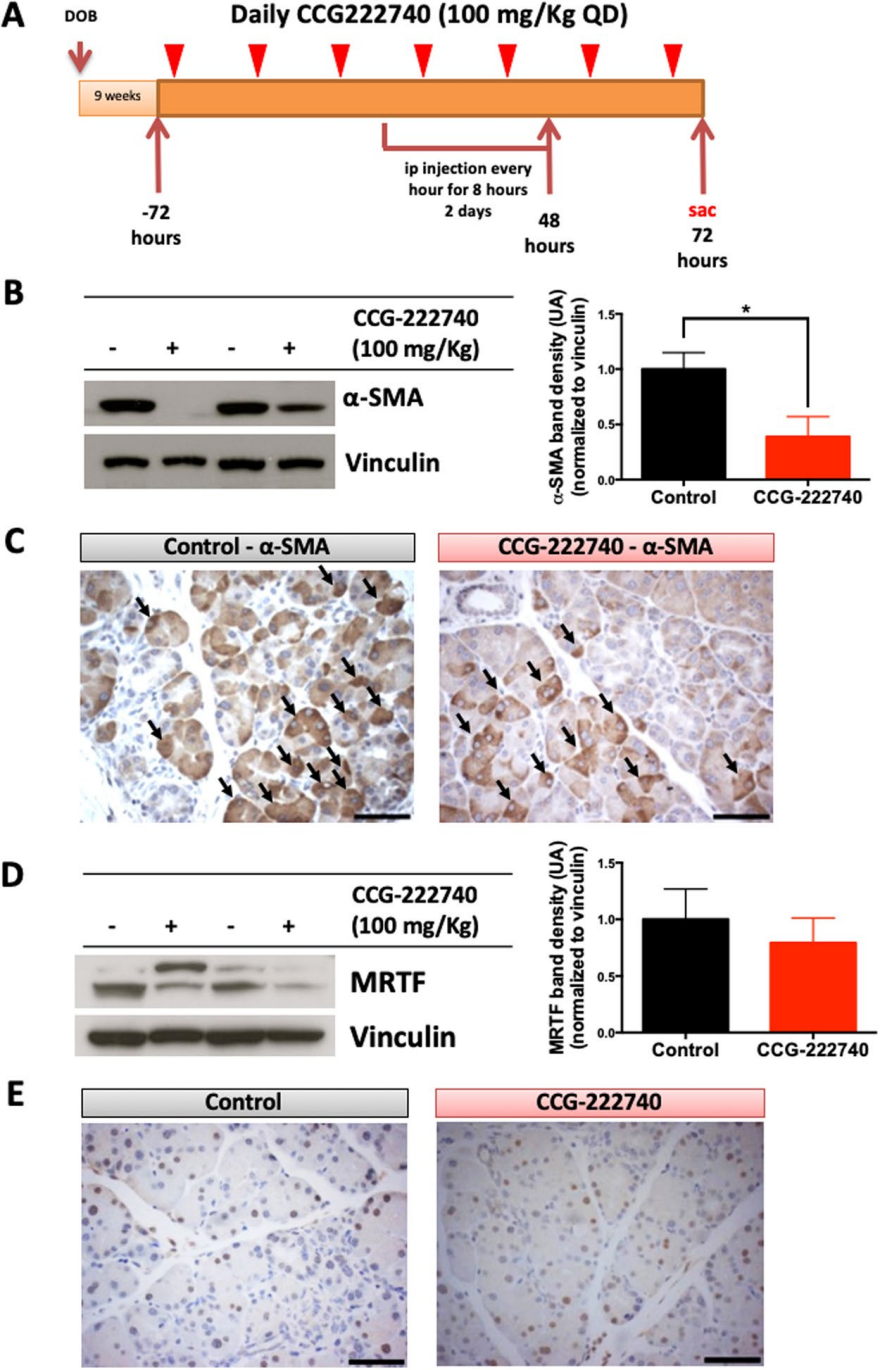
Rho/MRTF pathway inhibitor CCG-222740 decreases the levels α-SMA in KC mice stimulated with caerulein. (**A**) Experimental design, n = 7 mice/cohort. Nine-week old KC mice were randomized into 2 groups: group A – caerulein injections (75 μg/kg) were administrated over 2 days, one injection every hour for 8 hours with an overnight rest; group B was treated daily for 7 days with CCG-222740 (100 mg/kg by oral gavage), starting 72 hours prior to the caerulein challenge. All mice were sacrificed 72 hours after the last caerulein injection, and blood, pancreas and spleen were collected. (**B**) α-SMA levels were evaluated by western blot of pancreas lysates. Representative blots are shown for 2 animals per cohort (−control; +treated). Quantitation of α-SMA blots included all animals enrolled in the study (6 mice/cohort), and values were expressed as fold induction compared to caerulein stimulated controls. Full blots shown in Supplemental Fig. 12. (**C**) Expression of α-SMA in cells was detected by immunohistochemistry (scale bar: 60 μm). Arrows indicate stellate cells. (**D**) MRTF levels were evaluated by western blot of pancreas lysates. Representative blots are shown for 2 animals per cohort. Quantitation of MRTF blots included all animals enrolled the study (5 mice/cohort), and values were expressed as fold induction compared to caerulein stimulated controls. Full blots are shown in Supplemental Fig. 12. (**E**) Expression of MRTF in cells was detected by immunohistochemistry (scale bar: 60 μm). *p < 0.05, Caerulein 75 μg/kg vs CCG-222740 treatment.

Caerulein, a cholecystokinin analog, is a ten amino acid oligopeptide that stimulates smooth muscle contraction, increases digestive secretions, and is commonly used to induce either acute or chronic pancreatitis^30^. In mouse models of pancreatic cancer, caerulein promotes disease progression^27,31–33^. KC mice (LSL-Kras^G12D/+^; Pdx-1-Cre) at 9 weeks of age were pre-treated with CCG-222740 (100 mg/kg daily) by oral gavage, starting 3 days before caerulein stimulation. Treatment with CCG-222740 continued throughout the caerulein challenge and for an additional two days, for a total of 7 days. Caerulein was injected every hour for eight hours for 2 consecutive days (Fig. 4A). As shown by both western blotting and immunohistochemistry, CCG-222740 significantly reduced α-SMA levels in the pancreas of caerulein-stimulated KC mice (p < 0.05) (Fig. 4B,C, Sup Fig. 3). This downregulation occurred in the stellate cells surrounding the acinar structures (Fig. 4C, arrows point to PSCs), as confirmed by IHC. Levels of MRTF protein were not significantly altered by treatment with CCG-222740 in the KC mice (Fig. 4D). Immunohistochemistry demonstrated that MRTF is present in the nucleus of stromal, acinar and stellate cells, but no differences between the control and treated groups were observed (Fig. 4E). MRTF can be expressed in several cell types as a response to physiological stress, to induce mobility and cell to cell adhesions^34^.

### Effect of CCG-222740 treatment on immune cells in KC mice

Pancreatic stellate cells play a fundamental role in the process of disease promotion and progression by producing essential chemokines and cytokines that modulate the immune population in the pancreas^35^. CCG-222740 reduced the production of α-SMA, therefore less stellate cell activation was observed upon caerulein stimulation (Fig. 4B,C). This led us to hypothesize that pharmacological inhibition of the Rho/MRTF pathway with CCG-222740 would alter the immune compartment in the pancreas microenvironment.

**Figure 5:**
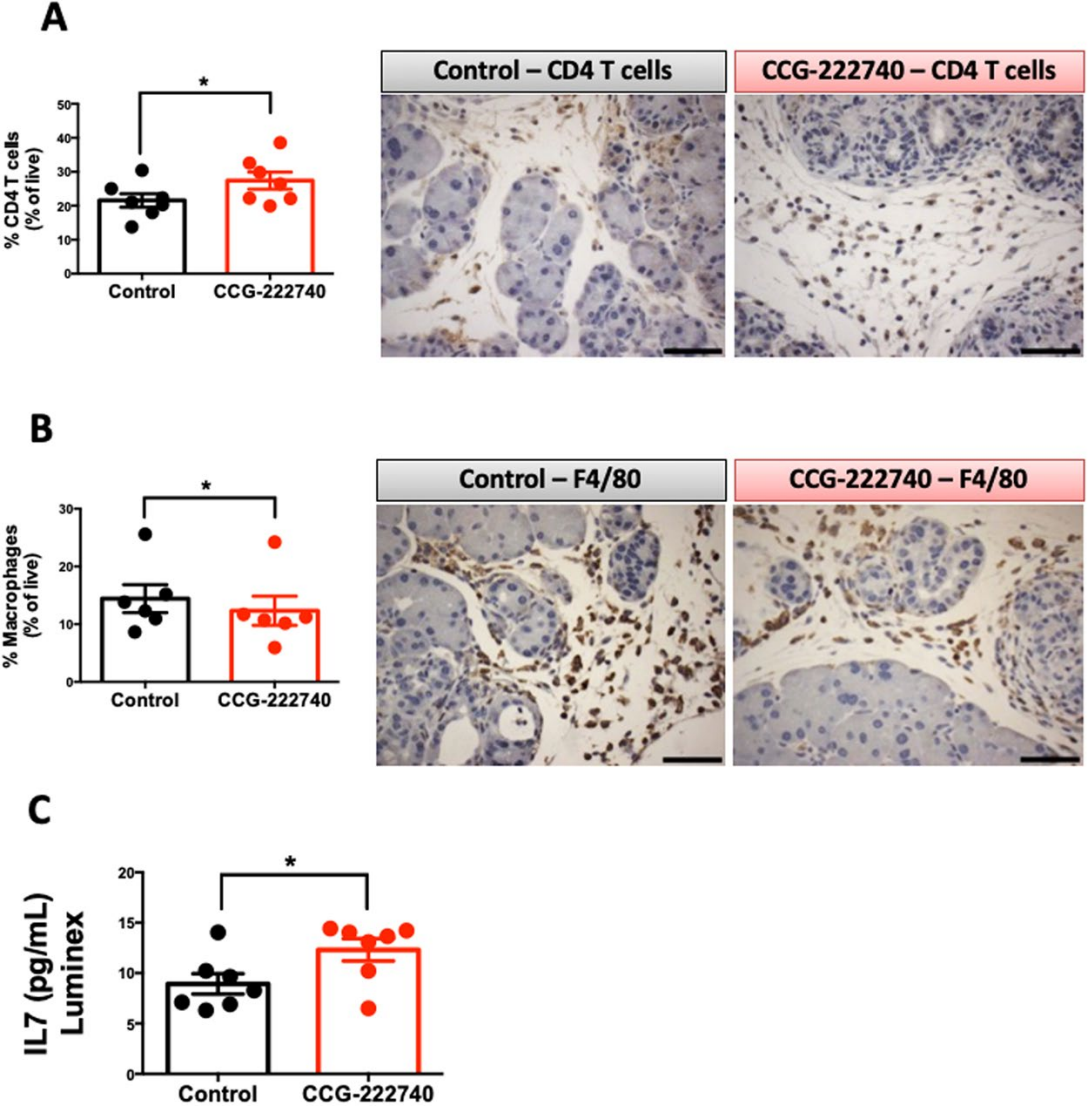
Rho/MRTF inhibitor CCG-222740 decreases the level of macrophages and increases CD4 T cells in the pancreas of KC mice stimulated with caerulein. Levels of CD45^+^, CD3^+^, CD4^+^ T cells (**A** - CD4 T cells, n = 7 per group) and CD45^+^, CD11b^+^, Gr1^−^ or F4/80^+^ cells (**B** – macrophages, n = 6 per group) were analyzed by flow cytometry and immunohistochemistry in the pancreas. Scale bar: 60 μm. (**C**) Levels of cytokines including IL-7 were determined by a cytokine multiplex assay in pancreas lysates (n = 7 per group). *p < 0.05, Caerulein 75 μg/kg vs CCG-222740 treatment.

KC mice develop pancreatic adenocarcinoma (PDA) over an extended period of time with simultaneous infiltration of immune cells. This time frame can be shortened with caerulein (Fig. 4A), which leads to an increase in the infiltration of immune cells, such as macrophages^27,36^. Because of the presence of mutant *Kras* in the ductal epithelial cells of the pancreas, infiltrating immune cells are skewed to support tumor progression^37,38^. KC mice treated with CCG-222740 showed a significant upregulation in the percentage of CD4 T cells (CD45^+^, CD3^+^, CD4^+^) infiltrating into the pancreas (p = 0.03), 21.6% in the control vs. 27.4% in the treated group (Fig. 5A). A significant downregulation in the percentage of macrophages (CD45^+^, CD11b^+^, Gr1^−^) in the pancreas of treated KC mice (12.2%) compared with the control mice (14.4%) was also observed (p = 0.03), as determined by flow cytometry and immunohistochemistry (Fig. 5B). Other immune cell populations were also analyzed, showing a significant increase in B cells by CCG-222740 treatment, but no changes in myeloid-derived suppressor cells or CD8 cytotoxic T cells (Sup Fig. 4). To test the anti-inflammatory properties of CCG-222740, RAW 264.7 macrophage-like cells were treated with this compound in the presence of LPS or INFγ. In both instances, the MRTF pathway inhibitor reduced the levels of NO, suggesting that CCG-222740 has anti-inflammatory properties (Sup Fig. 5).

Because CCG-222740 reduces pancreatic infiltration of macrophages and increases CD4 T cells and B cell infiltration (Fig. 5 and Sup Fig. 4), a cytokine multiplex assay was performed. Pancreas lysates and plasma of KC mice stimulated with caerulein (control group) and treated with CCG-222740 (treated group) were used for the cytokine multiplex assay. This assay allows exploration of the modifications induced by this small Rho/MRTF pathway inhibitor on the pancreas and plasma cytokine/chemokine milieu. The cytokine multiplex assay revealed that CCG-222740 significantly increased IL7 in pancreas lysates (Fig. 5C, Sup Fig. 6), but not in plasma (data not shown).

### Rho/MRTF pathway in human pancreatic cancer

**Figure 6:**
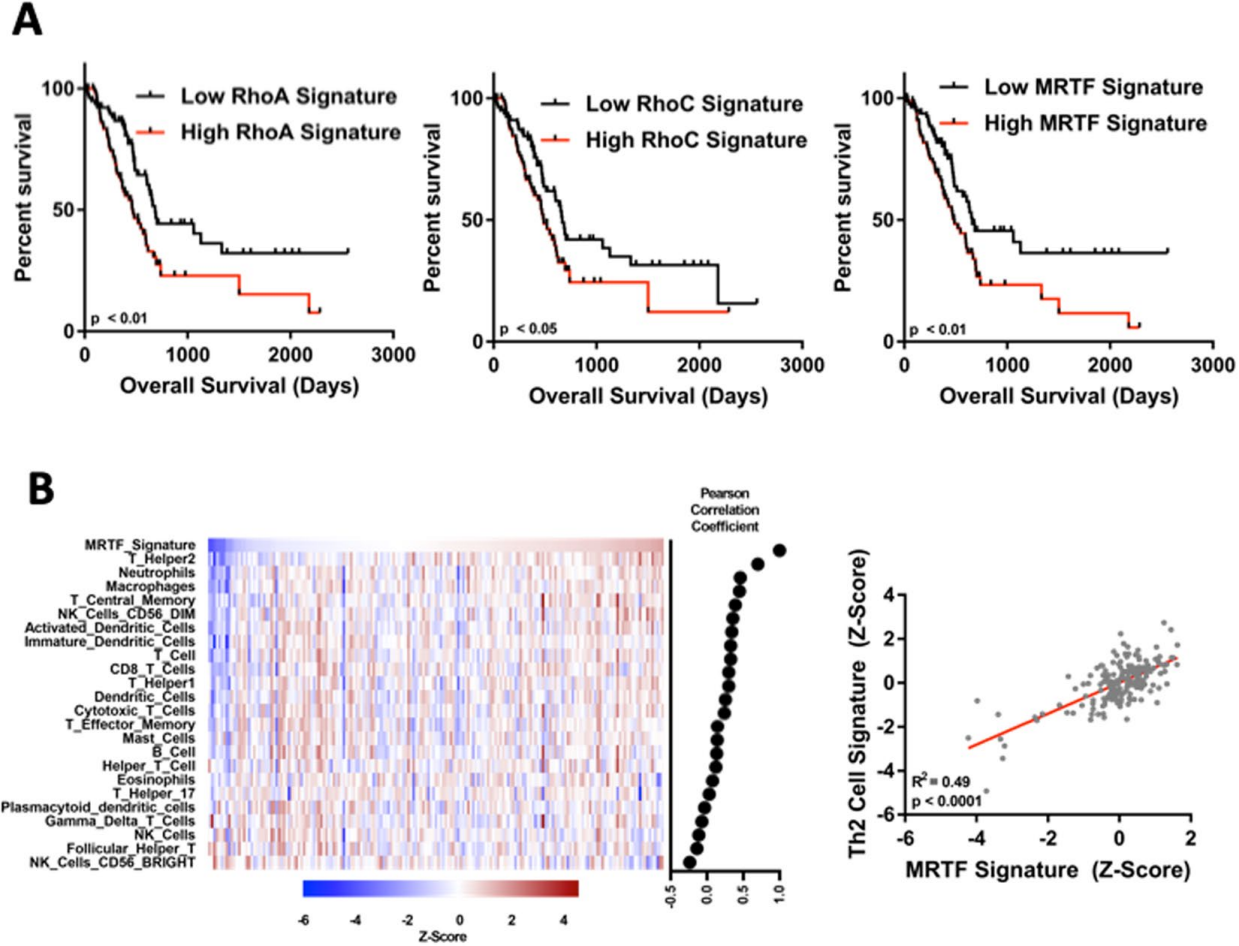
Rho/MRTF pathway expression in human pancreatic cancer. (**A**) The Cancer Genome Atlas (TCGA) pancreatic cancer dataset was stratified into halves based upon predicted RhoA, RhoC or MRTFA activation (n = 83 per group). Kaplan–Meier plots were generated from the highest (black) and lowest (red) expressing halves. Survival curves were analyzed with the log-rank (Mantel-Cox) test with a cutoff of P < 0.05 as statistically significant. (**B**) Pearson correlation between MRTF signature and the immune cell signatures. A MRTF gene signature correlates with a Th2 cell gene signature in the PDA TCGA dataset.

We next wanted to determine the clinical relevance of Rho and MRTF-induced gene expression in PDA. To do this, we tested for associations between Rho/MRTF gene expression signatures and overall survival in PDA patients using The Cancer Genome Atlas (TCGA) dataset. We have previously defined gene expression signatures for RhoA, RhoC and MRTF (10.1101/381806v2). Stratification of tumors by these RhoA, RhoC or MRTFA gene expression signatures in tumors with high expression (top half) and low expression (bottom half) shows a significant reduction in overall survival in patients with signatures of high RhoA, RhoC and MRTFA expression (Fig. 6A). However, high RhoB expression does not correlate with lower survival in PDA patients (Sup Fig. 7A). Moreover, gene signatures for RhoA and RhoC correlate with a MRTFA gene signature (Sup Fig. 7B).

The PDA tumor microenvironment is complicated by intercellular interactions between a multitude of different cell types, including immune cells, fibroblasts, and cancer cells. Because CCG-222740 alters immune cell infiltration in the KC mouse model, we tested whether MRTF pathway activation is correlated with immune cell infiltration in human tumors. Using data from the TCGA dataset, we observed that a MRTF signature is correlated with several immune cell populations, most notably with a Th2 cell signature (r = 0.49, p < 0.0001) (Fig. 6B).

## Discussion

Pancreatic cancer is characterized by a strong fibroinflammatory reaction composed mainly of cancer-associated fibroblasts. These cells are responsible for chemoresistance and help establish an immunosuppressive tumor microenvironment^2,6^. Pancreatic stellate cells, another important fibrotic cell type in the pancreatic stroma, play a crucial role in wound healing and stroma remodulation after injury^10,11^. Stellate cells support pancreatic tumor growth by supplying metabolic provisions and promoting an immunosuppressive milieu^7,35^. However, total ablation of the cancer-associated fibroblasts and stellate cells renders pancreatic tumors more aggressive^39^. These findings suggest that fibroblasts in the tumor microenvironment play both a tumor-promoting and a tumor-suppressive role. Thus, reprogramming CAFs so that they assume a tumor-suppressive role would likely be more beneficial than complete elimination of CAFs.

In our *in vitro* studies, the Rho/MRTF pathway inhibitor CCG-222740 suppressed stellate cell activation and subsequent production of pro-fibrotic ECM components by cancer-associated fibroblasts, without total elimination of these cells. In contrast, fasudil, a ROCK inhibitor increased the production of collagens in cancer associated fibroblasts. This finding suggests that targeting the Rho/ROCK/MRTF pathways at the level of transcription (MRTF inhibitors, CCG-222740) in CAFs might be more beneficial, since it decreased the production of collagens, important components of the ECM in pancreatic cancer^40^.

In KC mice stimulated with caerulein, stellate cells are activated; however, this activation is partially prevented by treatment with CCG-222740. In KC mice, the presence of an activating *Kras* mutation in combination with the inflammatory stimulus by caerulein results in faster development of pancreatic cancer^27,31^. The time frame used in the experiments reported in this manuscript do not allow the evolution of the precursor lesions to full adenocarcinoma, as described previously^27^. The work reported here focuses on the initial influence of the pancreas environment upon an inflammatory stimulus. Persistent activation of stellate cells leads to the formation of a fibrotic matrix that generates a physical barrier to therapy, preventing the diffusion of small molecules into the tumor. Modulating the fibrotic compartment of pancreatic cancer can have beneficial therapeutic implications when combined with other standard therapies, such as gemcitabine^13,41,42^. The data presented here suggest that CCG-222740 can modulate the activation of stellate cells in the context of *Kras* activation. Further studies are necessary to explore the effects of this compound at later time points and in an established fibrotic matrix, such as what is observed in established PDA tumors or after the development of fibrosis in a chronic pancreatitis model.

CCG-222740 was designed to inhibit Rho/MRTF-mediated gene transcription. Some components of this pathway were previously implicated in the promotion or progression of pancreatic cancer^43,44^. To our knowledge, no correlation between RhoA/C or MRTF and pancreatic cancer survival (Fig. 6A) has been reported previously. High expression of RhoA, RhoC and MRTFA gene signatures are negatively correlated with patient survival. These results highlight the importance of the Rho/MRTF pathway in pancreatic cancer survival and the need to develop effective small molecules that can successfully modulate this pathway.

MRTF functions as a transcriptional co-activator. CCG-222740 treatment of KC mice did not prevent the translocation of MRTFA to the nucleus (Sup Fig. 1C) or cause a reduction of the protein levels (Fig. 4D-E). However, the mechanism of action of CCG-222740 is complex (details described by Lisabeth *et al*.^45^). Additionally, MRTF is expressed in several cells in physiological conditions, including immune cells, which are responsible for cell mobility in response to environmental cues^34^. The lack of MRTF nuclear localization might be due to the fact that, at the time point used, caerulein induces an injury which causes infiltration of macrophages. As an important note, none of these immune cells (macrophages and T cells) have Kras mutations. The activation of MRTF is a physiological response to environmental cues, including to TGFβ, that has an important role in the development of the TME in pancreatic cancer^45,46^. Despite the retained MRTF nuclear localization, the levels of α-SMA were still effectively reduced by this small molecule (Fig. 4B,C). These observations suggest that CCG-222740 acts on the Rho/MRTF pathway, possibly by preventing the full assembly of transcription complexes that contain MRTFA and are dependent on the TGFβ signaling pathway. Similar reports have been described for other components of different transcription complexes, such as BRD4^47,48^. Moreover, the effects observed can also be due to inhibition by CCG-222740 of MRTFB, an alternative isoform of MRTF. Future studies will address these alternative hypotheses.

Fibroblasts and stellate cells produce several cytokines and factors that modulate the immune component in the pancreatic cancer microenvironment^13,35^. CCG-222740 reduced the infiltration of macrophages and increased the presence of CD4 T cells in the pancreas of caerulein-stimulated KC mice (Fig. 5A,B). Macrophages have been described as essential immune cells to promote pancreatic cancer development in the presence of *Kras* mutations^49,50^. Moreover, the presence of macrophages is associated with metastatic disease^51^, suggesting that CCG-222740 can be used to prevent the progression of pancreatic cancer. Additional ongoing studies are investigating the effects of CCG-222740 on macrophage polarization in the context of primary tumors and also in metastasis formation, since this compound can reduce the experimental metastasis of melanoma^22^.

High infiltration of CD4 T cells in pancreatic tumors correlates with extended survival^52^. Furthermore, a crosstalk between CD4^+^, GATA3^+^ T cells and cancer-associated fibroblasts was identified that resulted in greater infiltration of Th2 T cells and decreased patient survival^53^. In this study we show that CCG-222740 increases the infiltration of CD4 T cells into the pancreas. However, the short treatment times used in these experiments did not justify examining differences between T-bet^+^ and GATA3^+^ expression in CD4 T cells. The proportion of these two CD4 T cell populations is worth exploring in a long treatment protocol and/or in an established disease model after CCG-222740 treatment.

In our analysis of the cytokine multiplex assay for the pancreas lysates, IL7 was the only cytokine that was significantly altered by CCG-222740 treatment. IL7 is produced by stromal cells in the bone marrow and thymus and is critical for the development of all lymphocytes^54–56^. The role of IL7 in cancer is still not fully understood, however our findings suggest that the Rho/MRTF pathway may negatively regulate expression of this cytokine. Further studies are required to dissect the role of the Rho/MRTF pathway in cytokine segregation in stellate cells and recruitment of T cells to the pancreas. In this study we found no change in expression of cytokines associated with macrophage recruitment. However, macrophage recruitment and skewing toward a tumor promoting phenotype can also occur due to mechanical forces in the stroma^57^. The reduction in the infiltration of macrophages observed with CCG-222740 treatment may be due to reduction in the tension of the matrix, which was previously reported to be responsible for the recruitment of macrophages. Moreover macrophages are also responsible for the production of some matrix components, implying that macrophages may activate the Rho/MRTF pathway in order to produce essential matrix components and to maintain an immune suppressive phenotype^58^.

The experiments presented in this report suggest that the Rho/MRTF pathway may provide several points for targeting cells within the pancreatic cancer microenvironment. The findings provide evidence that the Rho/MRTF pathway is activated in the presence of *Kras* mutations, not only in stellate cells and cancer associated fibroblasts but also in some immune cells that may rely on this pathway to maintain their immunosuppressive phenotype. The role of the Rho/MRTF pathway in immune cells also requires further evaluation, since to our knowledge this is the first report to suggest the activation of this pathway in several immune cells.

The Rho/MRTF pathway has the potential to be an excellent drug target to modulate the pancreatic cancer stroma, thus making pancreatic tumors more amenable to traditional chemotherapy and immunotherapy. Further studies using a combination approach with CCC-222740 and currently approved drugs for the treatment of pancreatic cancer are warranted.

## Methods

### Drugs

CCG-222740 was synthesized as described^24^ by Scott Larsen at the University of Michigan. Compound purity was 94%.

### Cell culture

Murine cancer associated fibroblasts (CAFs)^6^ isolated from Kras LSL-G12D; Trp53 LSL-R172H; Pdx1-Cre (KPC) mice, a gift from Dr. David Tuveson (Cold Spring Harbor Laboratories, NY), and RAW 264.7 mouse macrophage-like cells (ATCC) were cultured in DMEM supplemented with 10% fetal bovine serum (FBS). Mouse pancreatic stellate cells (PSCs) were generated from wild type C57BL/6 mice by a modification of a method previously described^10^. Briefly, pancreatic tissue was minced with scissors and digested with Pronase 300 µg/mL (Roche, Indianapolis, IN), collagenase 750 µg/mL (Sigma Aldrich, St. Louis, MO), and DNase 8.75 µg/mL (Millipore-Sigma) in Gey’s balanced salt solution (GBSS; Sigma Aldrich) at 37 °C for 20 min. Digested tissue was then filtered through a 100 µm cell strainer (Becton, Dikinson and Company-BD Biosciences). Cells were washed once with GBSS, pelleted, and resuspended in 9.5 ml GBSS containing 0.3% bovine serum albumin (BSA) and 8 ml 28.7% Nycodenz solution (Sigma Aldrich; approximate density is 1.07). The Nycodenz solution containing the cells of interest was layered under 6 ml of GBSS containing 0.3% BSA, forming two phases, and centrifuged at 2000 rpm for 25 minutes at 4 °C. The cells of interest were harvested from the interface of the Nycodenz solution with the aqueous solution. Isolated PSCs were washed with GBSS and resuspended in DMEM containing 20% characterized FBS (HyClone), antibiotics (penicillin 100 U/ml and streptomycin 100 μg/ml, Invitrogen) glutamine, non-essential amino acids and sodium pyruvate. All cells were maintained at 37 °C in a humidified atmosphere of 5% CO_2_.

### Cell viability

Cells were seeded in 96-well plates at the following optimized densities: PanAsc 2159 (2500 cells/well)^59^, Aspc-1 (2000 cells/well) and CAFs (1500 cells/well). Cells were allowed to grow for 12 hours before serial dilutions of drugs were added. After 72 hours, cells were incubated with MTT (3-[4,5-dimethylthiazol-2-yl]-2,5- diphenyltetrazolium bromide; thiazolyl blue; Sigma-Aldrich; 5 mg/mL) for 4 hours. The supernatant was removed and developing solution (0.04 N HCl in isopropanol) was added. Plates were read at 630–570 nm.

### Cell cycle

Cell cycle was assessed by flow cytometry using a fluorescence-activated cell sorter (FACS-BD-LSRII). CAFs were treated with CCG-222740 at 10 µM for 72 hours, fixed in ice-cold 70% ethanol and treated with PI (Biolegend, 10 μg/mL)/RNase (Worthington, 0.1 mg/mL) solution. DNA content was quantified by flow cytometry.

### Western blotting

Cells treated with drugs were lysed in RIPA buffer (1 M Tris-Cl, pH 7.4, 0.5 M EDTA, 5 M NaCl, 1% triton-X, 25 mM deoxycholic acid, 0.1% SDS) containing protease inhibitors (PMSF, aprotinin and leupeptin). Pancreata were homogenized in EBC buffer (1 M Tris pH 8, 5 M NaCl) with the same protease inhibitors and 10% NP-40 and incubated on ice for 30 min. Protein concentrations were determined by the BCA assay (Sigma-Aldrich). Proteins were resolved by SDS-PAGE, transferred to a nitrocellulose membrane and analyzed with the following antibodies: vinculin and mouse and rabbit secondary antibodies (Cell Signaling); α-SMA, collagen I, collagen IV (Abcam); collagen 2 A (Santa Cruz, H-300). ImageJ was used to quantify the immunoblots, and results were plotted and statistically analyzed using Prism 6. All images shown are representative of 3 independent experiments.

### Inducible nitric oxide (NO) synthase assay

RAW 264.7 cells were plated in 96-well plates (20,000 cells/well), incubated with various concentrations of drugs, and stimulated with either 10 ng/ml of IFNγ (R & D Systems) or 1–3 ng/ml of lipopolysaccharide (LPS; Sigma-Aldrich, L4391) for 24 hours. NO levels in media were measured in the form of nitrite by the Griess reaction. I-BET 762 was used as a positive control.

### MRTF and Th2 signature

The Pancreatic Adenocarcinoma (PDA) TCGA RNA-Seq dataset was downloaded from the UCSC cancer genome browser (https://xenabrowser.net/datapages/) (accessed Jan. 2018). The MRTF gene signature is comprised of genes that are downregulated >2-fold upon MRTF knockdown in B16F2 melanoma cells^60^. The RhoA, RhoB, and RhoC gene signatures are described at 10.1101/381806v2, and the immune cell gene signatures are published^61^. The median expression of the immune cell signature genes was calculated for each sample in the TCGA dataset to produce a signature score. The MRTF signature was computed using ssGSEA on the genepattern server. Signature scores for each gene signature were z-score normalized across tumor samples, then the Pearson correlation coefficient was calculated between the MRTF signature and the immune cell signatures. All analysis was performed in R version 3.3.0.

### *In vivo* experiments

All animal studies were done in accordance with protocols approved by the Institutional Animal Care and Use Committee at Michigan State University. KC mice (LSL-Kras^G12D/+^; Pdx-1-Cre) were obtained by interbreeding male LSL-Kras^G12D/+^; Pdx-1-Cre and female Pdx-1-Cre mice. Genomic DNA was extracted from tail snips using the Extract-N-Amp tissue PCR kit (Sigma) and genotyped^36,62^. Four-week old KC mice were randomized and fed 5002 rodent chow. Nine-week old KC mice were treated with CCG-222740 (100 mg/kg) by oral gavage once a day for 7 days. CCG-222740 was dissolved in 15% (v/v) DMSO and then added to a solution of 10% tween-20 in saline. On the fourth day of treatment with CCG-222740, mice were injected intraperitoneally with caerulein (Sigma) at 75 μg/kg every hour for 8 hours for 2 consecutive days, with an overnight rest. Caerulein was dissolved in saline to the appropriate dilution.

### Flow cytometry

One third of the pancreas and spleen removed from KC mice was minced and incubated separately in digestion media consisting of collagenase (300 U/ml, Sigma), dispase (1 U/ml, Worthington), and DNAse (2 U/ml, Calbiochem) for 30 minutes at 37 °C with stirring. Cells were then passed through a 40 µm cell strainer (BD Falcon), and red blood cells eliminated with lysing solution. Single cells were resuspended in a solution of PBS/0.5% BSA/0.1% azide and stained for 30 minutes at 4 °C with the following antibodies: CD45-VioGreen (30F11, Miltenyi), Gr-1-PE (RB6-8C5, Miltenyi), CD11b-FITC (M1/70.15.11.5, Miltenyi), CD19-APC (1D3/CD19, Biolegend), B220-PerCP-Cy5.5 (RA3-6B2, Biolegend), CD3-PE (145-2C11, Biolegend), CD4-FITC (Gk1.5, Miltenyi), CD8-APC (53–6.7, Biolegend) and 5 μg/ml anti-mouse CD16/CD32 antibody (Biolegend) to reduce antibody binding to Fc receptors. Propidium iodide staining was used to exclude dead cells. Cells were analyzed using an LSR II-DIVA 6.2 software (BD) with three laser sources (488 nm, 633 nm, 407 nm) and FlowJo x.10.0.7r2 software (Tree Star).

### Immunohistochemistry

One third of the pancreas and spleen removed from KC mice was fixed in 10% phosphate-buffered formalin for 48 hours, embedded in paraffin blocks, and sectioned (5–6 µm). Hydrogen peroxide was used to quench endogenous peroxidase activity. Sections were immunostained with antibodies raised against α-SMA (1:50, 1A4, Abcam), F4/80 (1:40, BM8, EBioscience) or CD4 (1:40, Gk1.5, Biolegend), and visualized with biotinylated anti-rabbit or anti-rat secondary antibodies (Cell Signaling or Vector Labs). Signal was detected using a DAB substrate (Cell Signaling) following the manufacturer’s recommendations. Sections were counterstained with hematoxylin (Vector Labs). Human microarrays were acquired from US Biomax, Inc. (PA485 and T141a) and stained for α-SMA was described above.

### Multiplex cytokine assay

Plasma from KC mice was aliquoted and stored at −80 °C until use. Cytokine levels in plasma and pancreas whole lysates were measured using a Millipore mouse 32plex kit (EMD Millipore). Calibration curves from recombinant cytokine standards were prepared with threefold dilution steps. Standards and spiked controls were measured in triplicate, samples were measured once, and blank values were subtracted from all readings. Assays were carried out in a 96-well filtration plate (Millipore) at room temperature, following the manufacturer’s protocol. The fluorescence intensity of the multiplex beads was measured using a Luminex^®^ 200^TM^ array reader, and MAGPIX^®^ software with five-parametric-curve fitting was used for data analysis.

### Statistical analysis

Unless noted, all experiments were repeated at least three times, and representative images are shown. Results are described as mean ± standard error of the mean (SEM). Data were analyzed by t-test (Prism 6). All P values are two-sided, p < 0.05 was considered statically significant.

## Supplementary information

Supplemental Figures
