# Supplementary material for: The Rho/MRTF pathway inhibitor CCG-222740 reduces stellate cell activation and modulates immune cell populations in KrasG12D; Pdx1-Cre (KC) mice
Source: Sci Rep. 2019 May 8;9:7072. doi: 10.1038/s41598-019-43430-0 (PMC6506531; doi:10.1038/s41598-019-43430-0)
Supplement: Supplementary file 1 — Supplemental Figures [file 41598_2019_43430_MOESM1_ESM.pdf]

The Rho/MRTF pathway inhibitor CCG-222740 reduces stellate cell activation and modulates immune cell populations in $Kras^{G12D}$; Pdx1-Cre (KC) mice

Ana S. Leal, Sean A. Misek[1], Erika M. Lisabeth, Richard R. Neubig, Karen T. Liby

Authors' affiliations: Michigan State University, Department of Pharmacology & Toxicology, East Lansing, MI
[1]Department of Physiology

Corresponding author: Karen T. Liby, Michigan State University, Department of Pharmacology & Toxicology, B430 Life Science Building, 1355 Bogue Street, East Lansing, MI 48824. Phone: 517-884-8955; Fax: 517-353-8915; Email: liby.kare@msu.edu

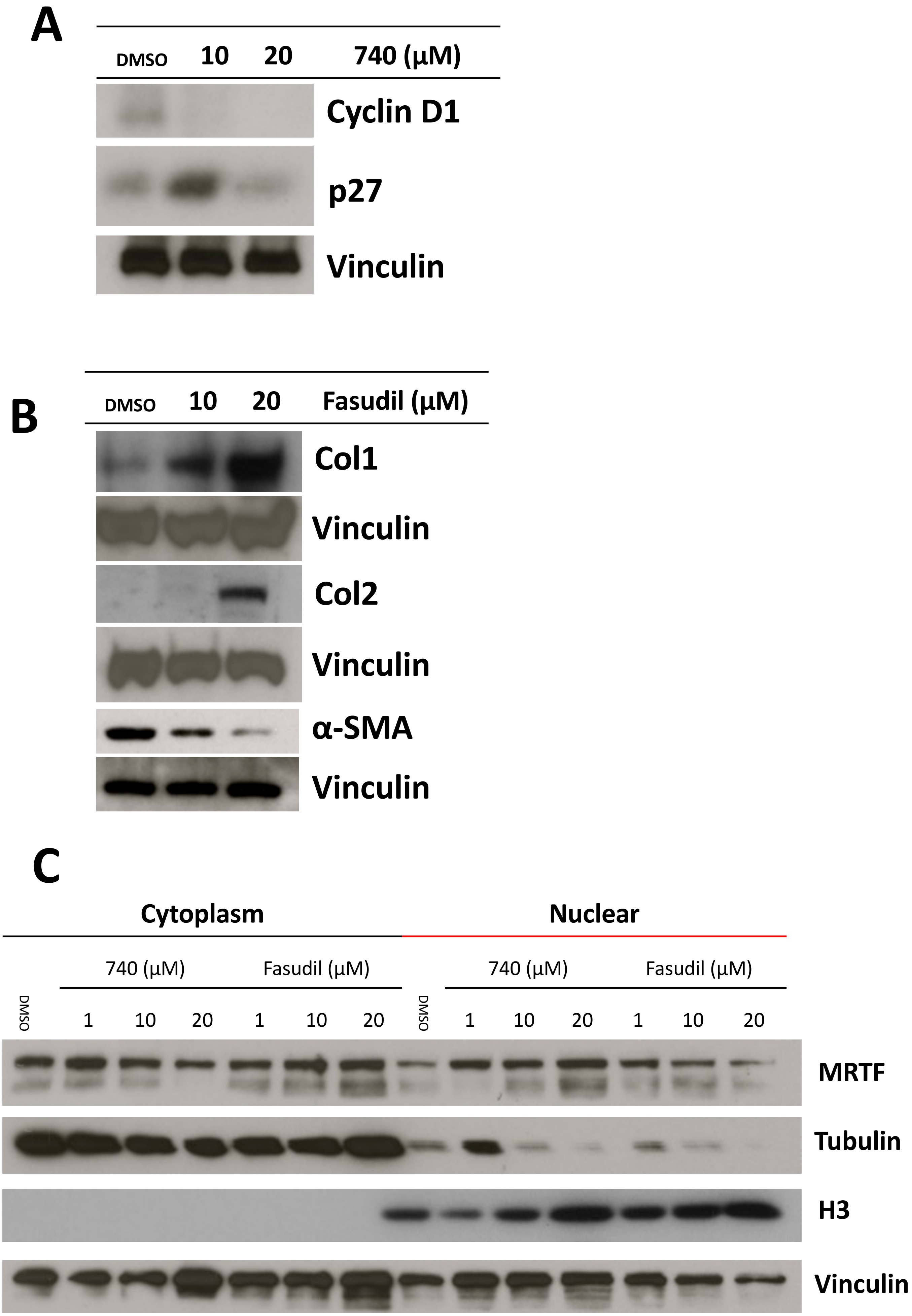
A
DMSO
10
20
740 (µM)
Cyclin D1
p27
Vinculin
B
DMSO
10
20
Fasudil (µM)
Col1
Vinculin
Col2
Vinculin
α-SMA
Vinculin
C
Cytoplasm
Nuclear
740 (µM)
Fasudil (µM)
740 (µM)
Fasudil (µM)
DMSO
1
10
20
1
10
20
DMSO
1
10
20
1
10
20
MRTF
Tubulin
H3
Vinculin

Supplemental Figure 1. Effects of CCG-222740 and Fasudil (Fas) in Pancreatic stellate cells and Cancer associated fibroblasts (CAFs). (A) CAFs were treated with several concentrations of CCG-222740 (740) for 72 hours, and levels of Cyclin D1 and p27 were evaluated by western blot. Additional and original western blot films on Supplemental figure 10A. **(B)** Protein levels of alpha smooth muscle actin (α-SMA), collagen 2A (COL2A), collagen I (COL I) and collagen IV (COL IV) were determined in CAFs after treatment with Fasudil for 72 hours. Additional and original western blot films shown in Supplemental figure 10B. **(C)** Nuclear versus cytoplasmic localization of MRTF-A in CAFs treated with CCG-222740 or Fasudil for 72 hours. MRTF-A localization was evaluated by western blot. Additional and original western blot films in Supplemental figure 11.

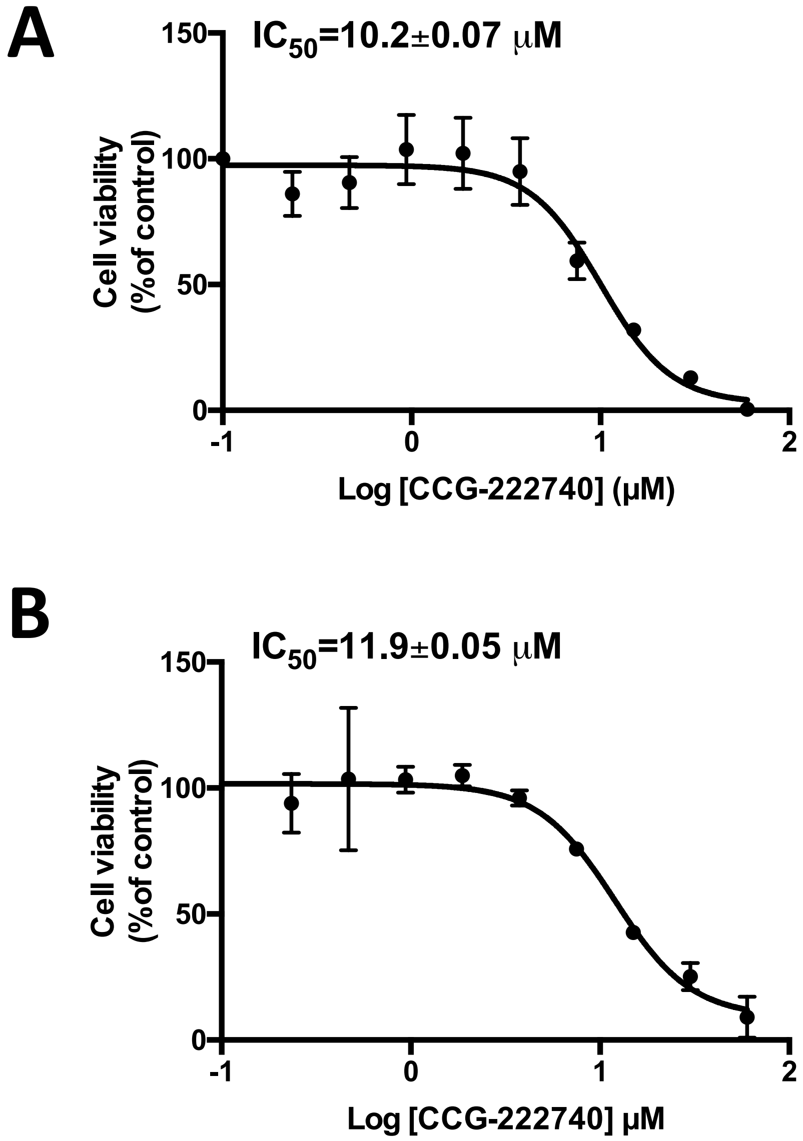

Supplemental Figure 2. CCG-222740 has growth inhibition effects on pancreatic cancer cells. Cells derived from mouse (PanAsc 2159) **(A)** and human (Aspc-1) **(B)** pancreatic tumors were treated with several concentrations of CCG-222740 for 72 hours. Cell viability was assessed by the MTT assay. The IC_{50} values shown are the mean ± SEM of 3 independent experiments.

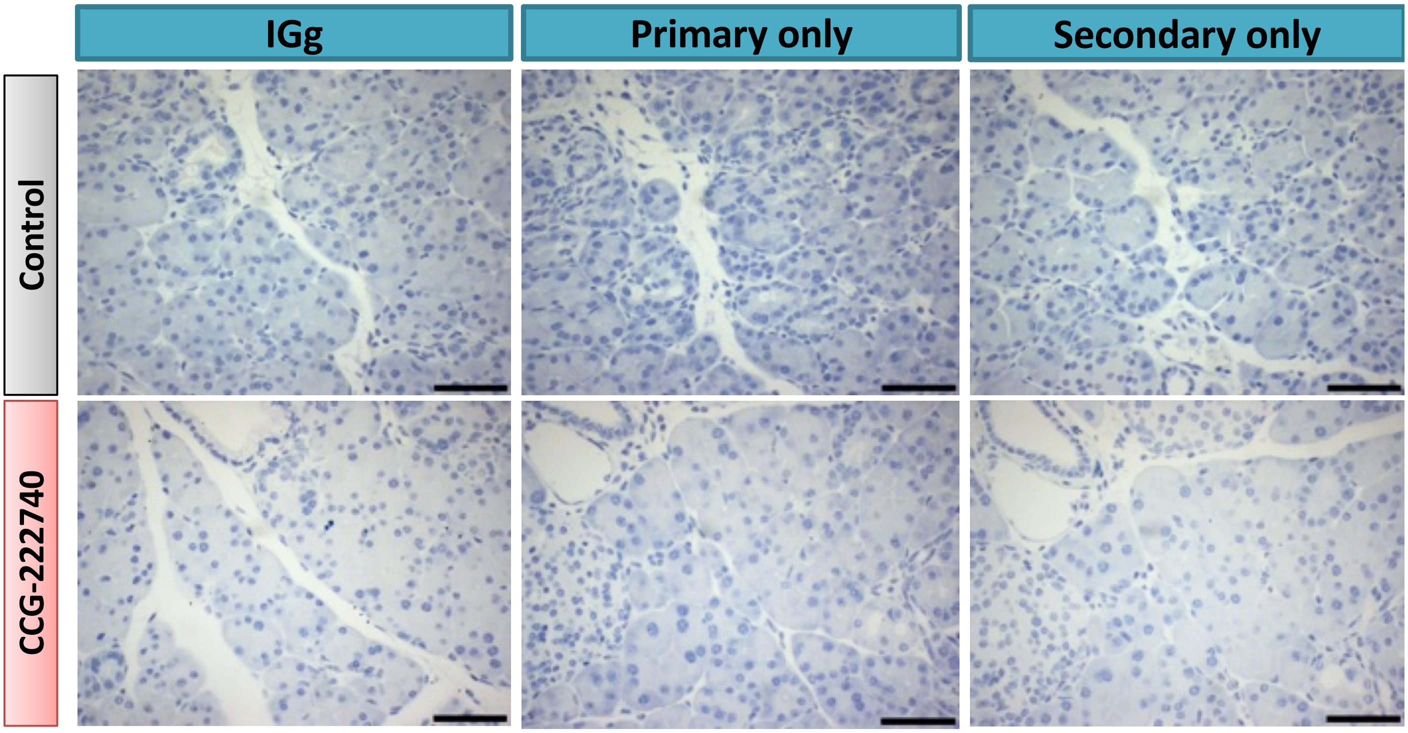

Supplemental Figure 3. Control staining for α-SMA. Control for non-specific staining in the pancreas of KC for α-SMA was done by using the same protocol for α-SMA but using IGg control, primary antibody only or secondary antibody only. Consecutive sections from the same samples were used for α-SMA staining. Scale Bar: 60 µm.

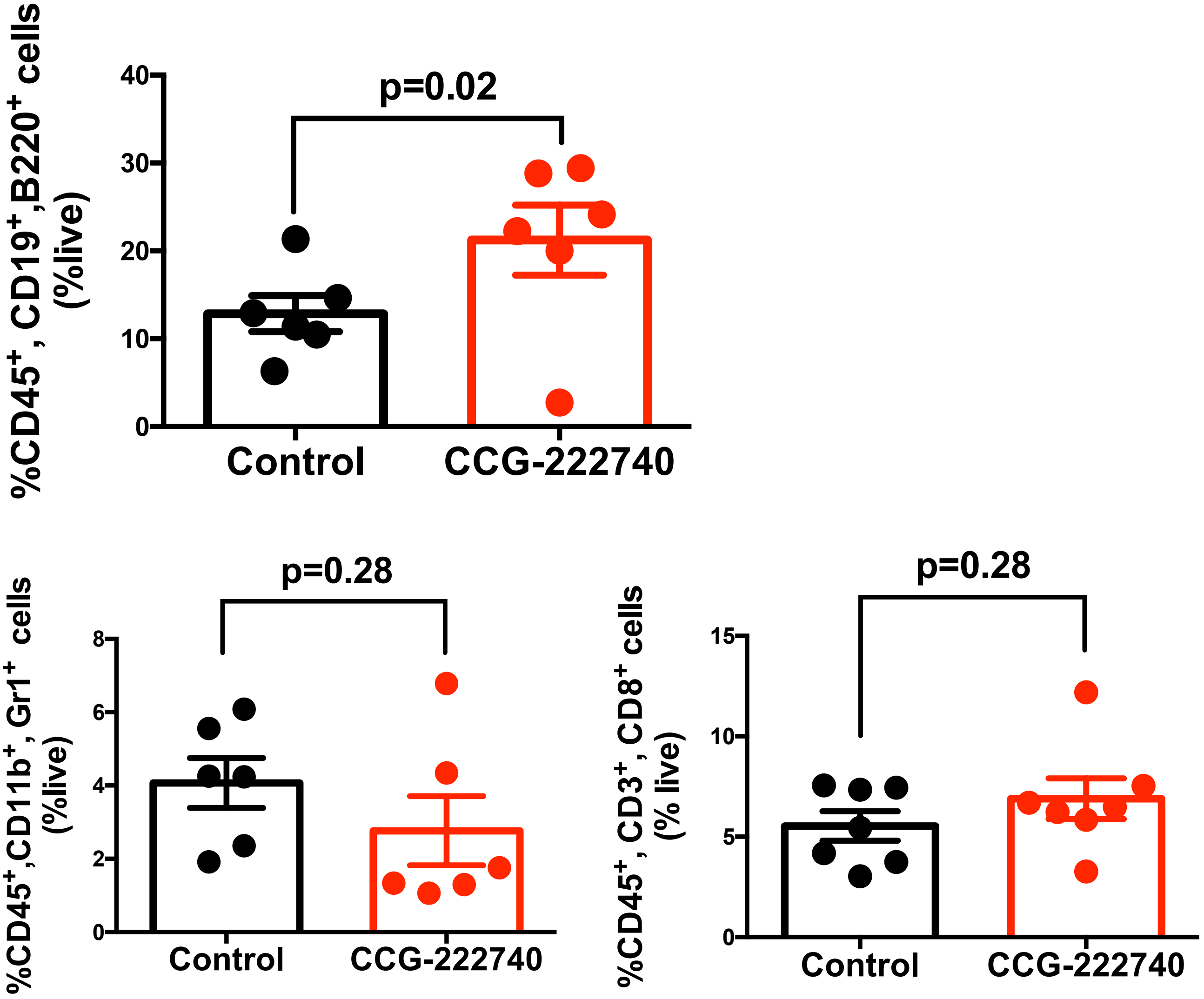

Supplemental Figure 4. MRTF inhibitor CCG-222740 increases the levels of B cells in the pancreas of KC mice stimulated with caerulein. Levels of $CD45^+$, $CD19^+$, $B220^+$ (B cells r, n=7) and $CD45^+$, $CD11b^+$, $Gr1^+$ cells (myeloid derived suppressor cells, n=6) and $CD45^+$, $CD3^+$, $CD8^+$ T cells (CD8 T cells) were analyzed in the pancreas by flow cytometry.

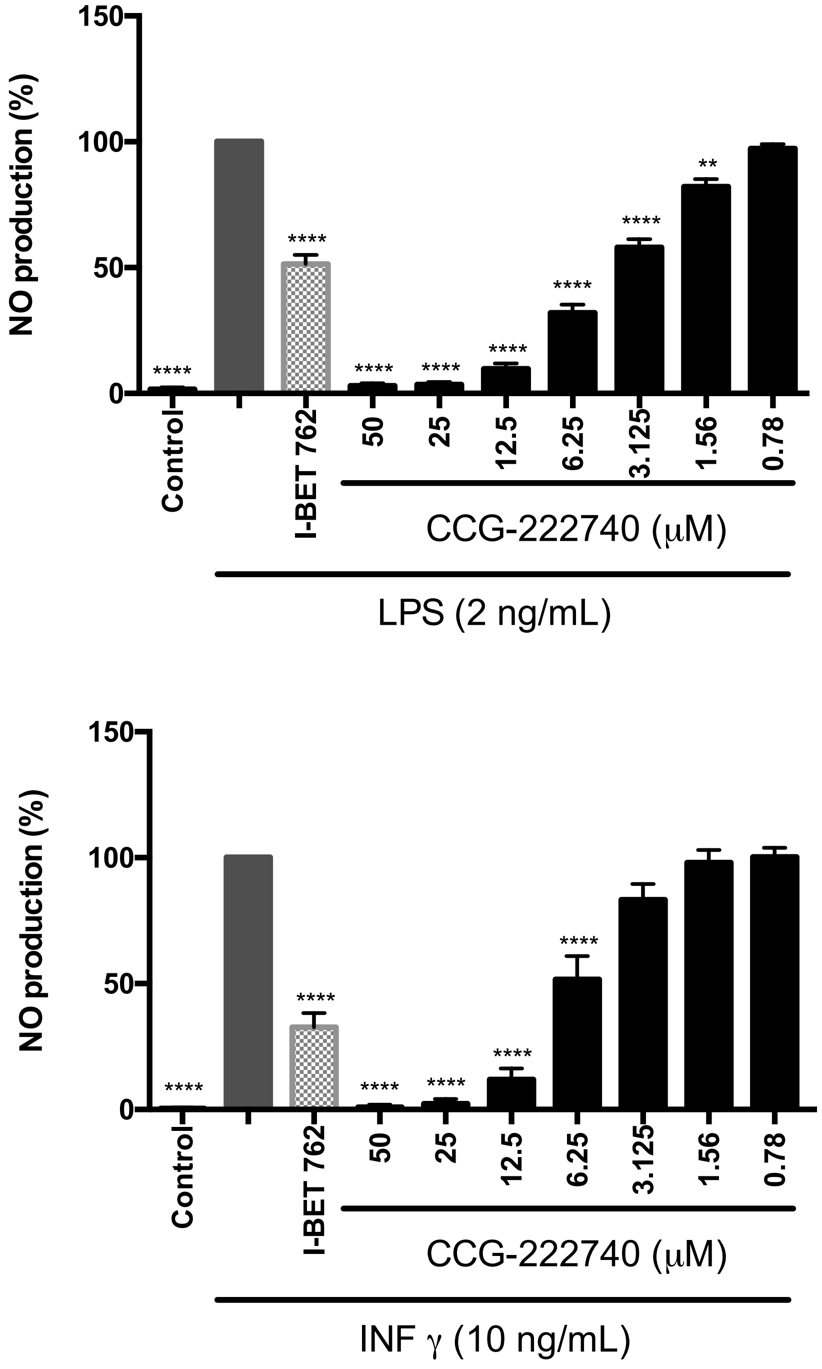

Supplemental Figure 5. CCG-222740 downregulates the production of NO *in vitro*. RAW 264.7 cells were pre-treated for 20 min with several concentrations of CCG-22740 and then stimulated with LPS (2 ng/mL) or INFγ (10 ng/mL) for 24 hours. Supernatants were collected and tested for NO levels using Griess reagents. I-BET 762 (250 nM) was included as a positive control. Mean and STD are shown for 4-6 independent experiments. * $p < 0.05$, ** $p < 0.001$, **** $p < 0.00001$ vs stimulated controls.

	Control							CCG-222740						
GCSF	11.6	10.3	13.3	6.1	9.2	7.9	5.1	9.2	16.7	7.9	10.6	9.2	6.8	6.8
EOTAXIN	439.4	371.3	270.8	369.8	802.3	452.1	164.5	578.5	116.5	296.9	1295.8	1189.0	392.1	1112.2
GM-CSF	73.0	55.2	26.4	48.0	78.3	52.6	25.5	57.8	13.7	35.3	71.5	134.8	21.6	105.4
IFNG	16.4	18.1	11.3	13.7	11.8	15.3	4.4	17.0	14.4	15.8	14.8	10.1	21.2	8.2
IL-1A	137.3	153.7	80.0	99.0	100.7	101.3	114.6	109.5	130.8	122.5	114.6	154.8	167.1	92.4
IL-1B	26.5	13.1	18.1	6.9	26.5	10.2	42.2	13.1	11.7	2.4	18.1	43.8	40.6	27.5
IL-2	24.0	25.9	22.3	18.5	21.3	19.9	15.9	22.4	19.9	24.5	22.1	24.0	24.9	17.1
IL-4	1.6	3.7	0.8	1.1	2.6	0.6	1.7	1.6	1.5	0.8	2.2	2.1	2.0	1.2
IL-3	1.9	1.7	1.9	0.9	1.4	1.5	0.3	1.5	2.3	1.5	1.4	1.7	1.2	0.8
IL-5	2.3	3.4	2.5	1.5	1.9	0.7	1.9	1.5	1.4	2.5	3.1	1.9	1.9	4.0
IL-6	16.0	12.3	10.8	10.5	15.4	17.0	6.1	13.0	16.0	12.7	15.2	13.5	12.3	12.1
IL-7	9.6	7.1	8.3	6.3	14.0	6.9	10.2	14.2	6.5	10.2	14.0	14.4	13.7	13.1
IL-9	293.3	336.5	239.4	232.1	263.7	227.3	313.2	260.3	266.0	307.0	280.4	271.5	348.3	285.8
IL-10	27.0	27.4	20.8	14.0	18.2	19.3	16.7	19.3	44.0	17.0	21.5	28.1	32.5	16.3
IL-12P40	29.8	24.7	23.7	24.7	26.1	33.1	17.6	36.8	40.7	24.2	28.9	28.9	27.5	19.1
IL-12P70	5.8	5.8	17.2	4.8	10.2	6.7	5.8	12.6	8.5	7.6	7.6	8.5	6.7	5.8
LIF	298.1	257.1	186.6	141.9	387.5	218.8	90.3	259.7	76.2	195.7	323.8	452.9	125.5	420.3
IL-13	118.4	124.5	118.4	96.9	127.5	118.4	258.8	129.0	121.5	106.2	127.5	133.4	118.4	115.4
LIX	71.6	70.2	87.6	68.7	77.6	79.2	70.4	87.4	74.5	70.9	79.2	82.9	75.4	72.6
IL-15	86.1	77.6	94.3	58.3	94.3	88.8	103.6	104.9	67.4	74.8	91.6	107.5	117.9	110.1
IL-17	1.1	2.1	0.7	0.5	0.8	1.3	1.0	1.3	1.4	0.7	1.7	1.1	11.1	1.5
IP-10	173.0	162.0	69.4	48.3	146.0	81.5	84.3	106.0	14.5	86.9	151.9	167.3	96.5	213.9
KC	851.6	659.4	451.9	319.5	708.8	610.2	321.2	723.2	138.0	404.8	810.2	581.2	611.7	1301.5
MCP-1	71.1	148.4	65.3	67.8	89.5	85.9	34.8	54.4	30.3	61.6	99.2	85.4	56.3	183.9
MIP-1A	73.4	94.0	66.7	66.7	87.9	70.7	60.5	73.4	77.0	55.0	72.9	84.0	72.3	80.8
MIP-1B	21.1	25.4	17.9	18.4	22.6	18.9	17.9	19.2	16.8	17.5	19.9	22.6	17.9	25.9
M-CSF	35.2	31.2	33.5	30.0	40.3	34.7	35.2	39.2	18.7	18.7	39.8	51.2	33.5	46.9
MIP-2	118.9	125.1	112.2	105.1	122.0	115.6	164.5	115.6	141.8	115.6	128.0	133.8	154.2	139.2
MIG	105.5	124.7	97.5	51.1	210.1	90.7	180.5	79.4	30.2	71.3	134.9	233.4	355.0	163.7
RANTES	27.6	30.5	21.6	17.9	45.3	22.8	30.2	23.2	20.3	24.2	30.8	28.8	40.0	31.1
VEGF	100.9	70.4	311.7	26.2	185.7	147.4	101.9	180.1	171.5	51.5	156.6	431.2	166.5	188.1
TNFA	3.9	2.8	2.5	1.7	3.7	3.0	1.3	1.7	2.1	1.9	3.9	3.0	1.7	6.0

Supplemental Figure 6. MRTF pathway inhibitor CCG-222740 alters cytokine/chemokine (s) levels in the pancreas of KC mice stimulated with caerulein. Levels of cytokines/chemokines were determined by a multiplex assay in pancreas lysates (n=7 per group).

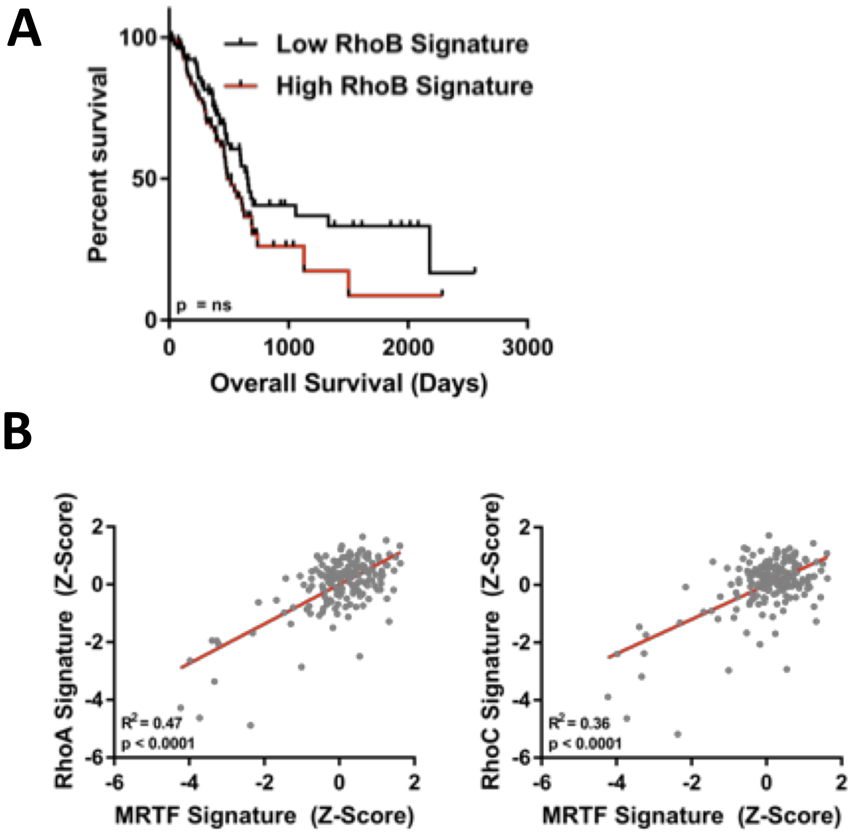

Supplemental Figure 7. RhoB/MRTF pathway expression in human pancreatic cancer. (A) The Cancer Genome Atlas (TCGA) pancreatic cancer dataset was stratified into quartiles based upon expression of RhoB (n=83). Kaplan–Meier plots were generated from the highest (black) and lowest (red) expressing quartiles. Survival curves were analyzed with the log-rank (Mantel-Cox) test with a cutoff of $P < 0.05$ as statistically significant. **(B)** MRTF gene signature correlates with RhoA and RhoC genes in data extracted from the TCGA data set for pancreatic cancer. Pearson correlation between MRTF signature and the RhoA and RhoC signatures.

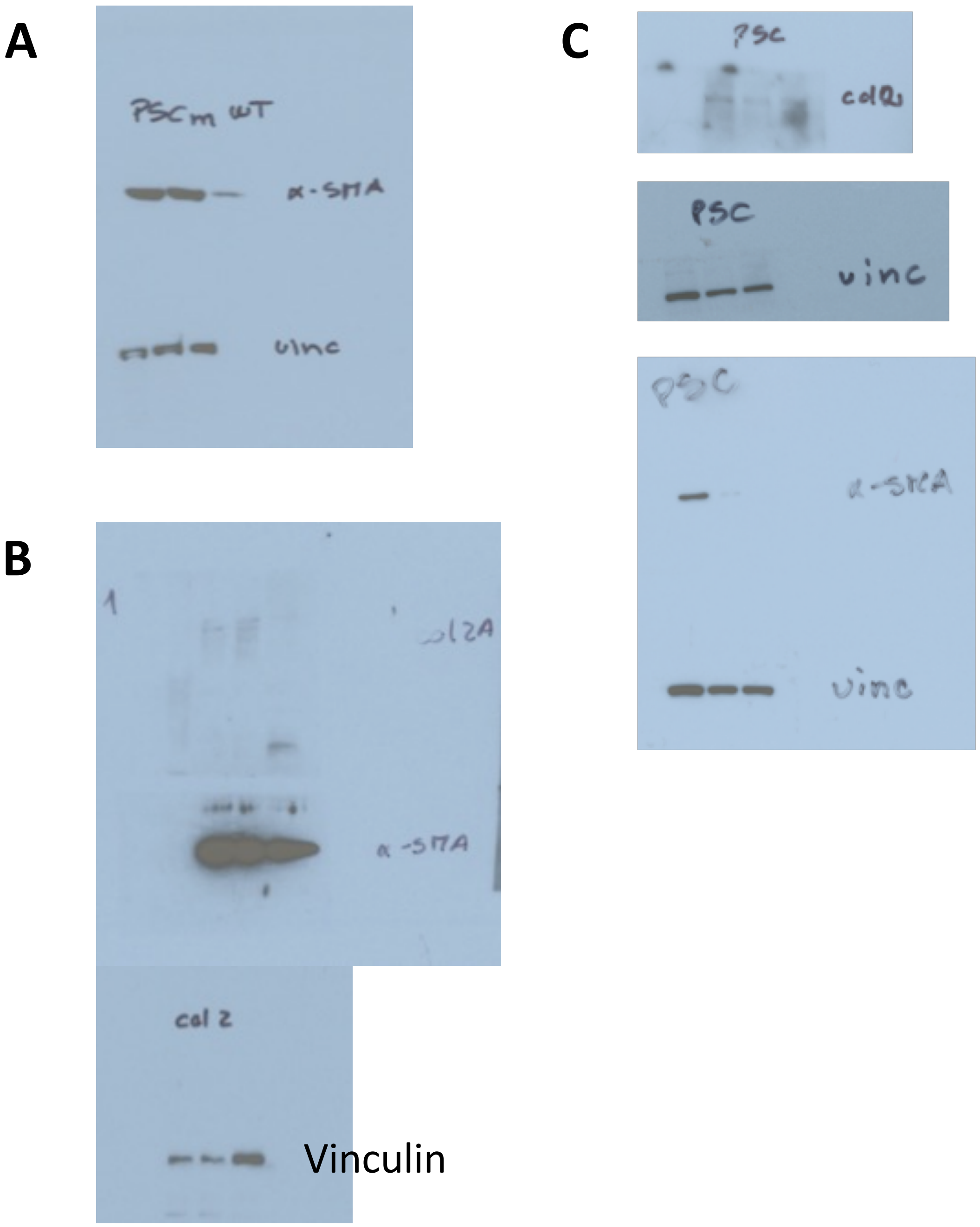

Supplemental Figure 8. Examples of immunoblots for treatment of mouse stellate cells with I-BET 762, Fasudil and CCG-222740. (A) αSMA and vinculin (loading control) probed in the same blot. (B) Collagen 2A and αSMA probed in the same blot, membrane for collagen 2A was reprobed for vinculin. (C) Collagen 2A, membrane was reprobed for vinculin, in PSCs treated with CCG-222740 and fasudil.

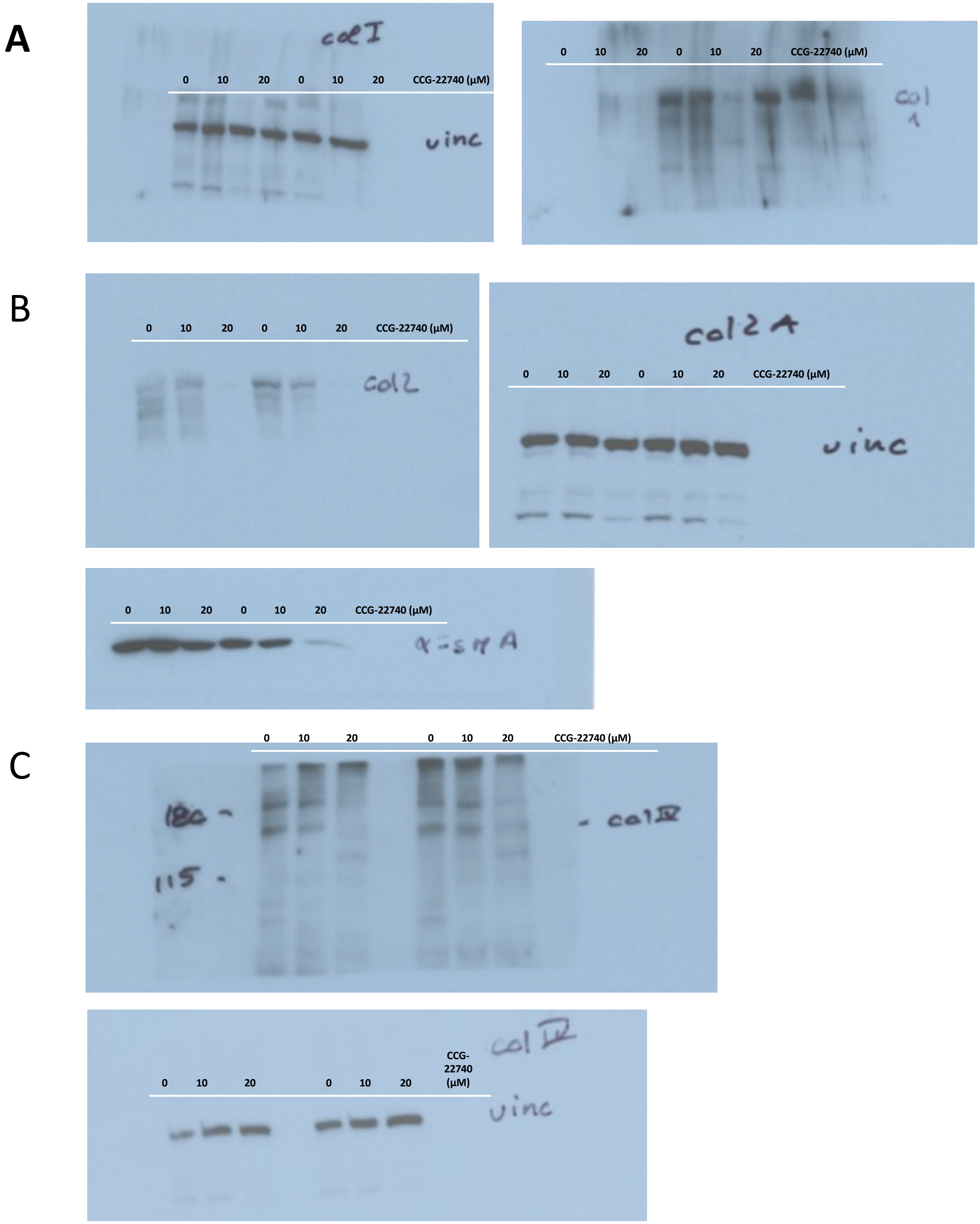

Supplemental Figure 9. Examples of immunoblots for treatment of mouse CAFs with several concentrations of CCG-222740. Two independent treatments were probed in the same blot. (A) Collagen I, vinculin was used as a loading control and the same membrane as for collagen I. (B) Collagen 2A and αSMA , vinculin was used as a loading control and the same membrane as for collagen 2A. (C) Collagen IV, vinculin was used as a loading control and the same membrane as for collagen IV.

A

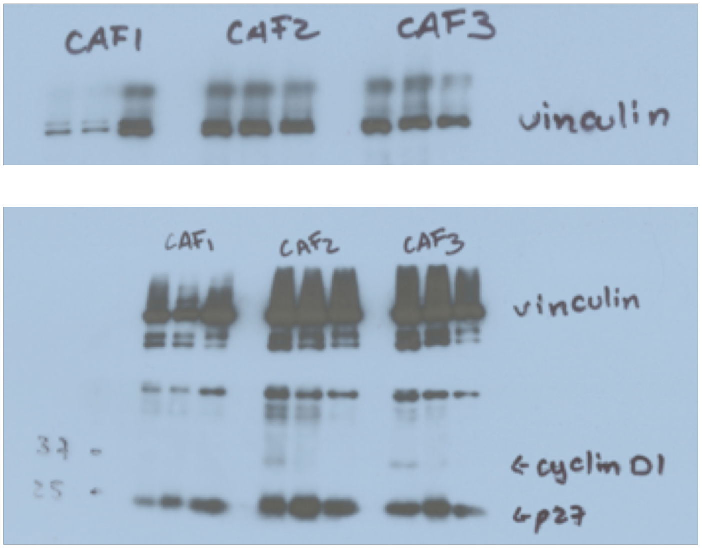

B

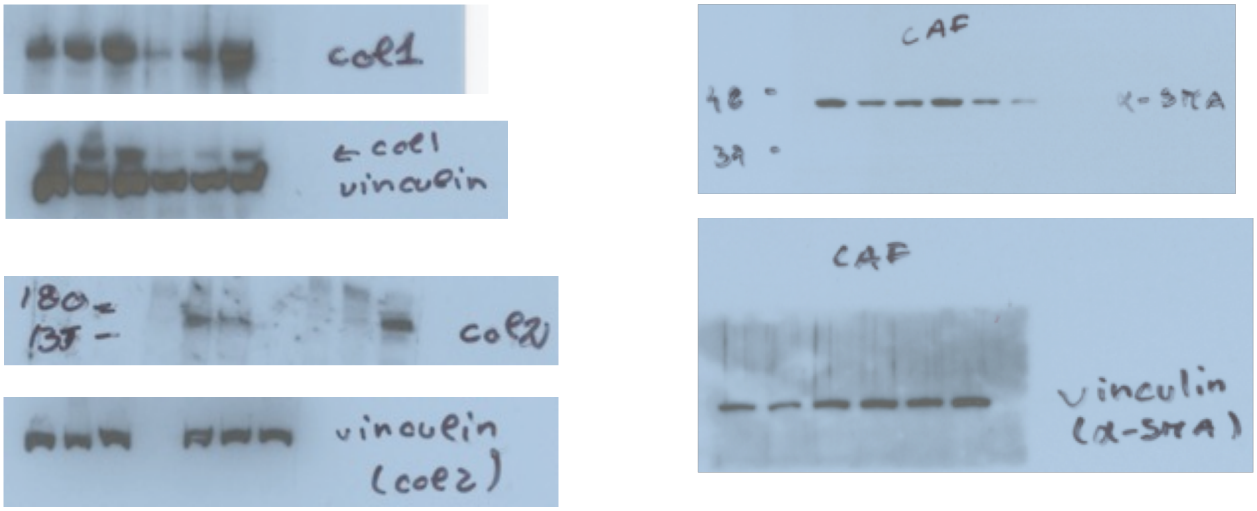

Supplemental Figure 10. Examples of immunoblots for treatment of mouse CAFs with several concentrations of CCG-222740 or Fasudil. Two-three independent treatments were probed in the same blot. (A) Cyclin D1 and p27 levels for CAFs treated with CCG222740 for 72 hours (B) Levels of Collagen I and 2 in CAFs treated with Fasudil for 72 hours. Collagen I, vinculin was used as a loading control and the same membrane as for collagen I. Collagen 2A and vinculin was used as a loading control and the same membrane as for collagen 2A.

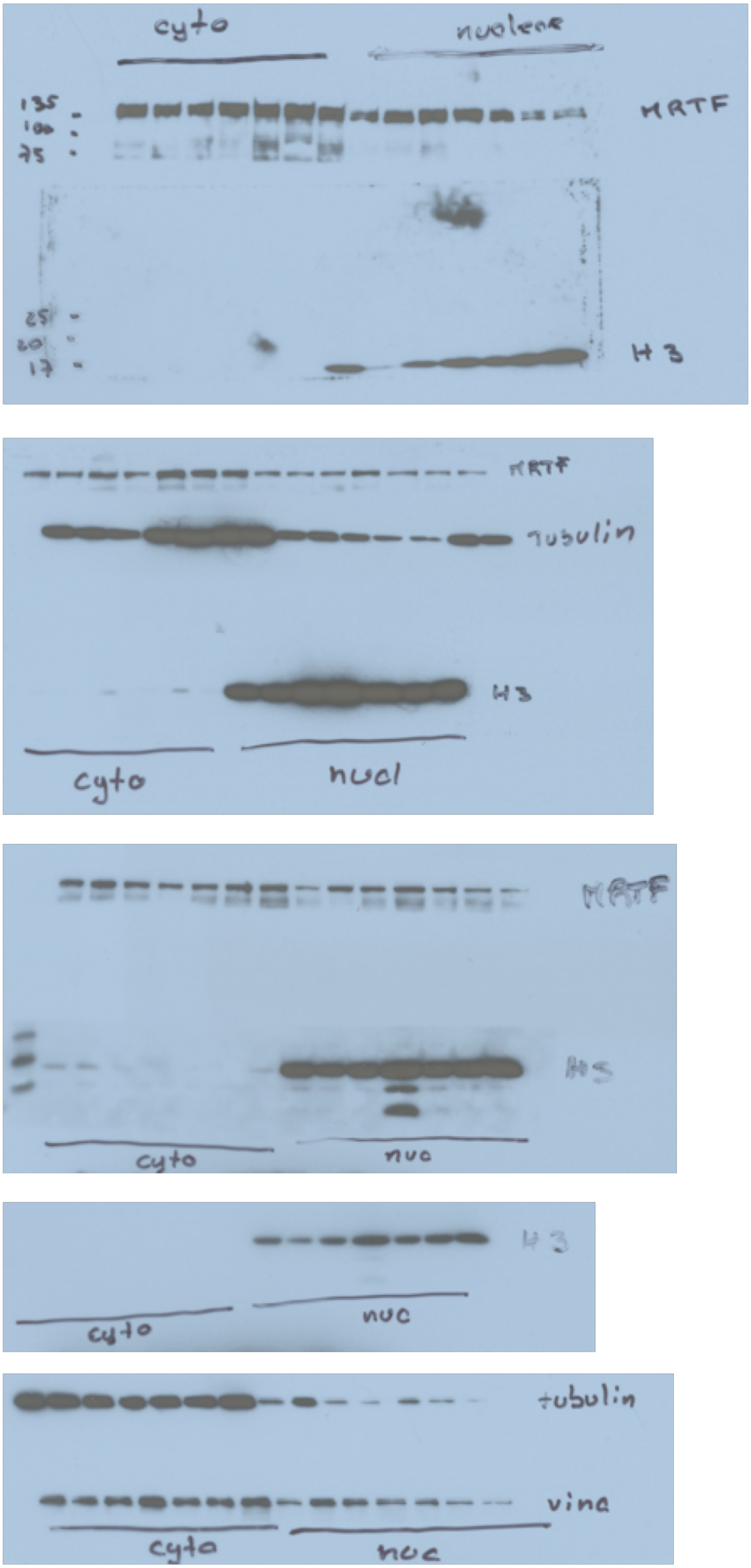

Supplemental Figure 11. Examples of immunoblots for cytoplasmic and nuclear localization of MRTF after treatments of CAFs with Fasudil and CCG-222740.

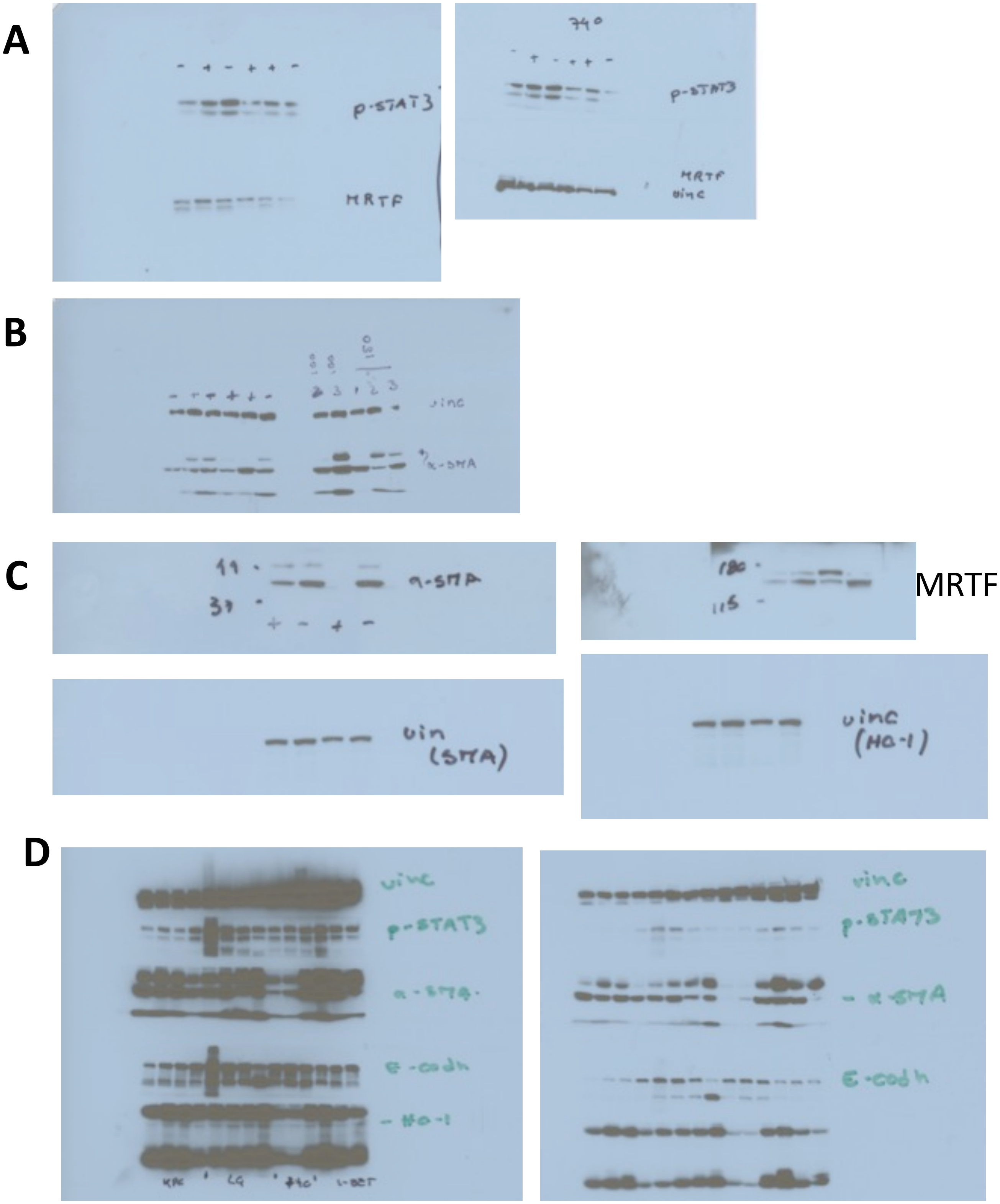

Supplemental Figure 12. Immunoblots used in the quantification of SMA and MRFT expression in the pancreas of KC mice treated with CCG-222740 (n=7, +) or vehicle (n=7, -). Levels were evaluated as the mice were enrolled on the study. (A) Membranes were probed for p-STAT3 and MRTF, them stripped and reused to probe vinculin. (B) Membranes were used to probe αSMA and vinculin simultaneously. (C) Membranes were probed for a series of proteins, shown are the blots for αSMA and MRTF, membranes were reprobed for vinculin. (D) A series of treatments was used in KC mice, pancreas extracts were used to probe levels of several proteins. αSMA and vinculin were used to quantify the CCG-22740 (740) treatment. Two different exposures are shown.
